# Intrinsic RIG-I restrains STAT5 activation to modulate antitumor activity of CD8^+^ T cells

**DOI:** 10.1172/JCI160790

**Published:** 2023-05-01

**Authors:** Xinyi Jiang, Jian Lin, Chengfang Shangguan, Xiaoyao Wang, Bin Xiang, Juan Chen, Hezhou Guo, Wu Zhang, Jun Zhang, Yan Shi, Jiang Zhu, Hui Yang

**Affiliations:** 1 Shanghai Institute of Hematology, State Key Laboratory of Medical Genomics, National Research Center for Translational Medicine at Shanghai, Ruijin Hospital, Shanghai Jiao Tong University School of Medicine, Shanghai, China.; 2 Center for Tumor Diagnosis & Therapy, Jinshan Hospital, Fudan University, Shanghai, China.; 3 Department of Oncology, Ruijin Hospital, Shanghai Jiao Tong University School of Medicine, Shanghai, China.; 4 Key Laboratory of Computational Biology, Shanghai Institute of Nutrition and Health, University of Chinese Academy of Sciences, Chinese Academy of Sciences, Shanghai, China.; 5 Collaborative Innovation Center of Hematology, Ruijin Hospital, Shanghai Jiao Tong University School of Medicine, Shanghai, China.

**Keywords:** Immunology, Oncology, Colorectal cancer, Immunotherapy, T cells

## Abstract

Antitumor activity of CD8^+^ T cells is potentially restrained by a variety of negative regulatory pathways that are triggered in the tumor microenvironment, yet, the exact mechanisms remain incompletely defined. Here, we report that intrinsic RIG-I in CD8^+^ T cells represents such a factor, as evidenced by observations that the tumor-restricting effect of endogenous or adoptively transferred CD8^+^ T cells was enhanced by intrinsic *Rig-I* deficiency or inhibition, with the increased accumulation, survival, and cytotoxicity of tumor-infiltrating CD8^+^ T cells. Mechanistically, T cell activation–induced RIG-I upregulation restrained STAT5 activation via competitive sequestering of HSP90. In accordance with this, the frequency of RIG-I^+^ tumor-infiltrating CD8^+^ T cells in human colon cancer positively correlated with attenuated survival and effector signatures of CD8^+^ T cells as well as poor prognosis. Collectively, these results implicate RIG-I as a potentially druggable factor for improving CD8^+^ T cell–based tumor immunotherapy.

## Graphical abstract

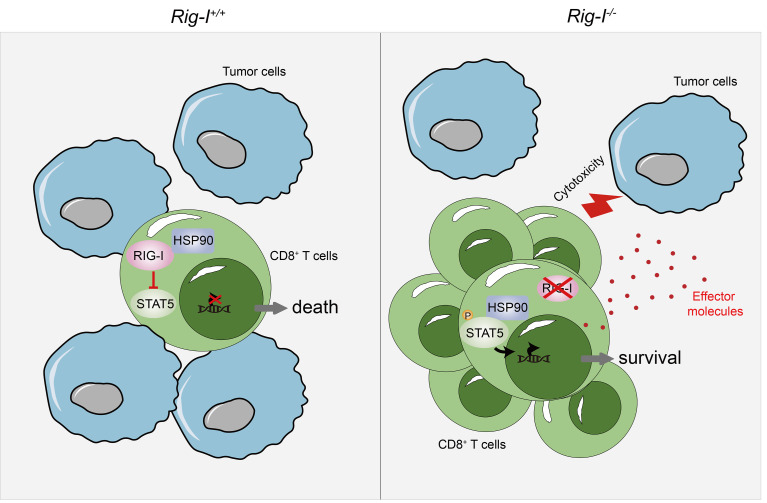

## Introduction

Tumor-infiltrating CD8^+^ T cells that specifically recognize tumor antigens act as an important type of immune effector and/or regulatory cell in direct control of cancer lesions (1). The binding of T cell receptor to MHC class I molecule–presented antigenic peptides plays a primary role in triggering the activation and differentiation of naive CD8^+^ T cells, which is otherwise critically regulated by a variety of costimulatory and coinhibitory signaling pathways (2). The activated CD8^+^ T cells are characterized by the enhanced synthesis and release of cytotoxic molecules such as perforin and granzymes, also named as cytotoxic T lymphocytes (CTLs). Activated CD8^+^ T cells also produce a variety of other cytokines (including IFN-γ, TNF-α, IL-2, and more) to create an inflammatory microenvironment that facilitates the activation, proliferation, survival, and functions of immune cells, including CTLs, while restricting the survival and proliferation of tumor cells (3). Nevertheless, negative regulatory mechanisms have been inherently incorporated into the responsive program of CD8^+^ T cells, which is required for maintaining immune homeostasis after clearing invading pathogens and for ensuring self-immune tolerance. In fact, these negative regulatory mechanisms have been exploited by tumor cells and the tumor microenvironment (TME) to evade immunorejection. Among them, activation-induced cell death (4) and functional exhaustion (1) have attracted attention from researchers when designing strategies to boost CD8^+^ T cell-based immunotherapy. For example, immune checkpoint blockade is designed to block the enhanced coinhibitory signaling pathways in the activated CTLs — such as those inhibiting programmed cell death protein-1 (PD-1) and cytotoxic T-lymphocyte antigen-4 (CTLA-4) — so as to reinvigorate CTL attacks on tumors (5, 6). Similarly, agonist antibodies or small molecules that augment T cell functions via other signaling pathways have also come into clinical application (7, 8). In addition, great efforts have focused on improving the infiltration and functionality of adoptively transferred T cells in the TME (9), yet, critical mechanisms involved in regulating the survival, retention, and effector function of these tumor-infiltrating CD8^+^ T cells remain poorly understood.

Nucleic acids are major structures detected by the innate immune system. The best characterized pattern recognition receptors (PRRs) that recognize nucleic acids in the cytosol of mammalian cells are RIG-I like receptors (RLRs) and cGAS–STING pathways, which detect RNA and DNA species, respectively (10). RNA ligand priming triggers multiple signaling transduction cascades that converge on the transcriptional activation of type I interferons (11). In the context of tumor immunotherapy, RIG-I within tumor and innate immune cells is primarily studied, as T cells are insensitive to RNA transfection (12, 13). Double-stranded RNA (dsRNA), an exogenous RIG-I agonist, works as an adjuvant that potently promotes tumor cell death (14, 15) and activates murine and human dendritic cells (16–18). dsRNA administration has also been found to synergize with immune checkpoint blockade to induce antitumor immunity (19, 20). On the other hand, the cGAS-STING pathway, as a dominant pathway that responds to cytosolic DNA in the context of tumor immunity, cellular senescence, and inflammatory diseases (21), has been well studied in T lymphocytes (22, 23). For example, it was reported that substantial cell death experienced by tumor-infiltrating T cells is in part mediated by IFN-independent activities of STING (24). Other than that, RNA-unprimed RIG–I also plays active roles in tumor settings (25). We and others identified that intrinsic and unprimed RIG-I in tumor and innate immune cells mainly exists as an antitumor molecule, as the expression of RIG-I reduces the proliferation and stemness maintenance of leukemia progenitor cells (26) and improves the antitumor effects of IFN-α therapy in hepatocellular carcinoma patients (27). However, insights into a possible intrinsic role of RIG-I in tumor-infiltrating CD8^+^ T cells have lagged significantly behind.

Whole-body *Rig-I* deletion in mice results in colitis-like autoimmune diseases with a decrease in naive T cells and an increase in effector T cells (28). The autoimmune phenotype of *Rig-I*–deficient mice is reminiscent of immune checkpoint receptor–knockout mice (29, 30). Further analysis showed that intrinsic RIG-I exerts a distinct role in Treg/Th17 cell balance, at least, in part, by restraining STAT3 activation (31). These findings suggest that loss of *Rig-I* may result in better survival and a hyperactivated state of T cells, thus promoting antitumor immunity. Here, we used *Rig-I^–/–^* mice, among other models, to demonstrate that intrinsic RIG-I regulates CD8^+^ T cell survival and antitumor cytotoxic function. Activation of CD8^+^ T cells in vitro upregulated the expression level of *Rig-I*, which led to cell death and compromised cytotoxicity, while tumor-bearing mice transferred with *Rig-I*-deficient CD8^+^ T cells exhibited delayed tumor growth. Mechanistically, elevated RIG-I associated with and deprived HSP90 from association with STAT5, thereby restraining STAT5 activation. In line with this, analysis of public databases indicated that an increased *RIG-I* mRNA level in tumor-infiltrating CD8^+^ T cells was associated with more enriched apoptosis and a less-enriched cytotoxic gene signature. Importantly, translational studies using surgical samples of intestinal cancer confirmed that the expression of *RIG-I* in CD8^+^ T cells was significantly increased in advanced patients, which may contribute to poor survival. Taken together, our study reveals a previously unappreciated function of RIG-I in CD8^+^ T cells in the context of antitumor immunity.

## Results

### Extratumoral Rig-I ablation inhibits tumor growth by enhancing CD8^+^ T cell–mediated immunosurveillance.

**Figure 1:**
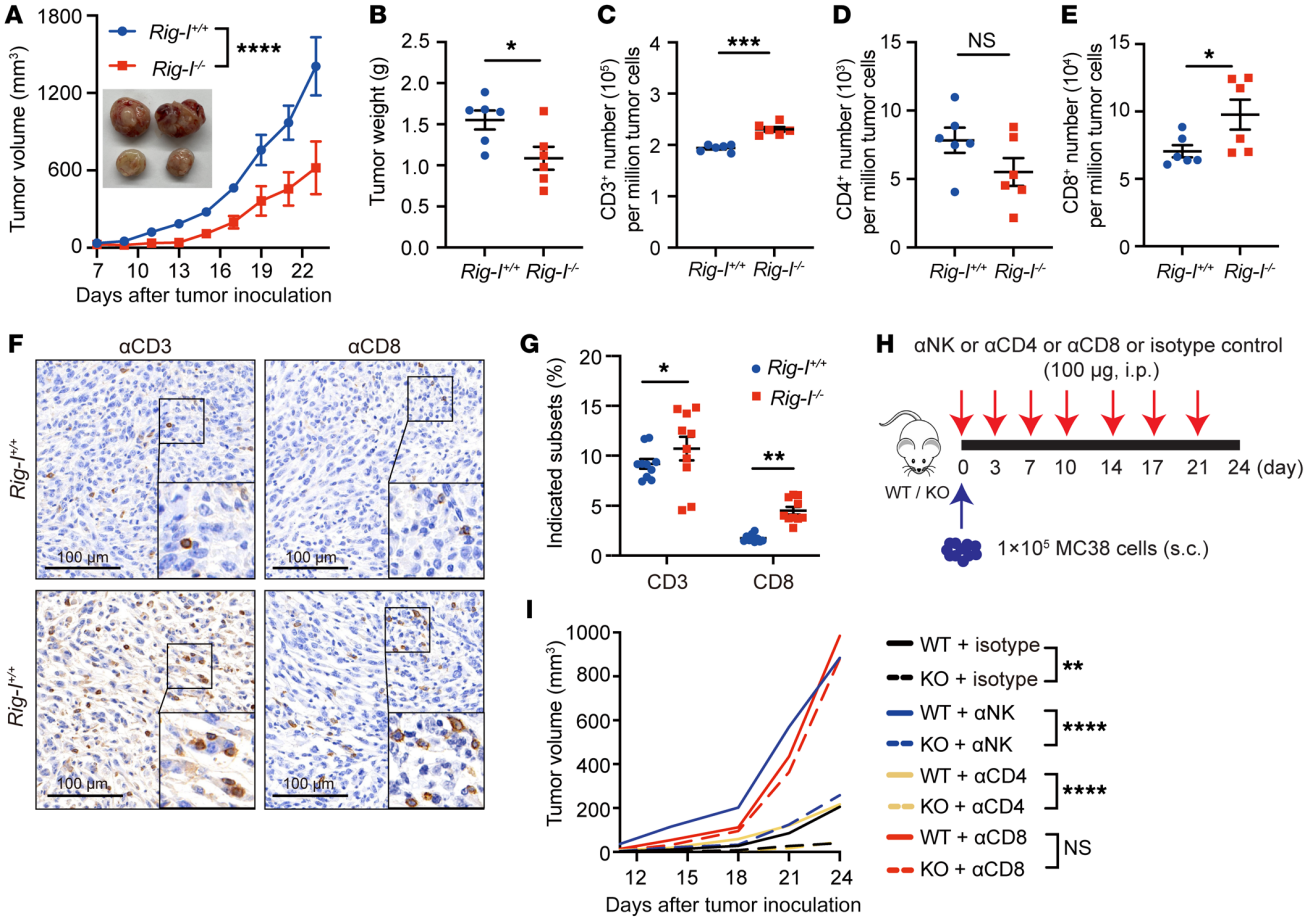
*Rig-I* ablation inhibits tumor growth mainly through CD8^+^ T cells. (**A** and **B**) 2 × 10^5^ MC38 cells were s.c. inoculated into 6-week-old *Rig-I^+/+^* or *Rig-I^–/–^* mice and the tumor growth was monitored for 27 days. (**A**) Tumor growth curves were plotted after measuring tumor size every 2 days beginning on day 7, and representative images of tumors retrieved from tumor-bearing mice on day 27 are shown in the inset. (**B**) On day 27, tumors retrieved from *Rig-I^+/+^* or *Rig-I^–/–^* mice were weighed (*n* = 6 per group). (**C**–**E**) Quantification of indicated immune cell subsets from dissociated MC38 tumors in *Rig-I^+/+^* or *Rig-I^–/–^* mice. (**F** and **G**) Representative IHC staining images of MC38 tumor tissue (**F**) or combined plot of percentages of indicated cell subsets (**G**). 10 random fields of view for each group were counted. (**H** and **I**) 1 × 10^5^ MC38 cells were s.c. inoculated into 6-week-old *Rig-I^+/+^* or *Rig-I^–/–^* mice and anti-CD4, anti-CD8, anti-NK1.1 antibody or isotype IgG control antibody were i.p. injected into tumor-bearing mice on days 0, 3, 7, 10, 14, 17 and 21. Tumor volume was measured every 3–4 days beginning on day 9 (*n* = 5 per group). Data are representative of at least 2 independent experiments and expressed as mean ± SEM. *P < 0.05, ***P < 0.001, ****P < 0.0001, by 2-way ANOVA (**A** and **I**) or unpaired Student’s *t* test (**B**–**E** and **G**).

To explore the potential function of extratumoral RIG-I in regulating immunosurveillance against tumors, we s.c. injected murine colon carcinoma (MC38), lymphoma (EL4) or melanoma (B16F10) cells into syngeneic C57BL/6 WT (*Rig-I^+/+^*) or *Rig-I^–/–^* (KO) mice. To avoid active autoimmune diseases confounding our analyses, 6-week-old *Rig-I^+/+^* or *Rig-I^–/–^* mice were used, as under the age of 8–10 weeks, C57 background *Rig-I^–/–^* mice looked as healthy as *Rig-I^+/+^* littermates, with similar spleen sizes and apoptotic/activated level of splenic CD8^+^ T cells (Supplemental Figure 1, A–E; supplemental material available online with this article; https://doi.org/10.1172/JCI160790DS1). Notably, *Rig-I* ablation significantly hindered tumor growth in all 3 inoculated tumor models (Figure 1, A and B, and Supplemental Figure 1F). Flow cytometric analysis of MC38 tumor-infiltrating immune cells showed an increased accumulation of CD8^+^ T cells, but not CD4^+^ T cells, NK1.1^+^, or CD19^+^ cells in the *Rig-I^–/–^* mice compared with their WT littermates (Figure 1, C–E, and Supplemental Figure 1G), which was verified by IHC analysis of tumor-infiltrating T cells (Figure 1, F and G). Suggestive of a crucial involvement of CD8^+^ T cells in restraining tumor growth within *Rig-I^–/–^* mice, antibody-mediated depletion of CD8^+^ T cells,but not CD4^+^ T or NK1.1^+^ cells, eliminated the restriction on tumor growth specifically by the *Rig-I^–/–^* host, while NK cell depletion promoted tumor growth similarly in *Rig-I^+/+^* and *Rig-I^–/–^* mice (Figure 1, H and I, and Supplemental Figure 1, H and I). Likewise, adoptively transferred *Rig-I^+/+^* or *Rig-I^–/–^* NK cells delayed tumor growth to the same level (Supplemental Figure1, J–L). Since it was reported that the deficiency of the mitochondrial antiviral signaling protein (MAVS), the essential partner of RIG-I in transducing the classic RLR pathway, reduced the intracellular STAT1 level, proliferation, survival, and cytotoxicity of NK cells (32), our observations indicate that the antitumor cytotoxicity of CTL, but not NK cells, is affected by RIG-I when functioning in a way independent of the RNA ligand-induced classic RLR activation pathway.

**Figure 2:**
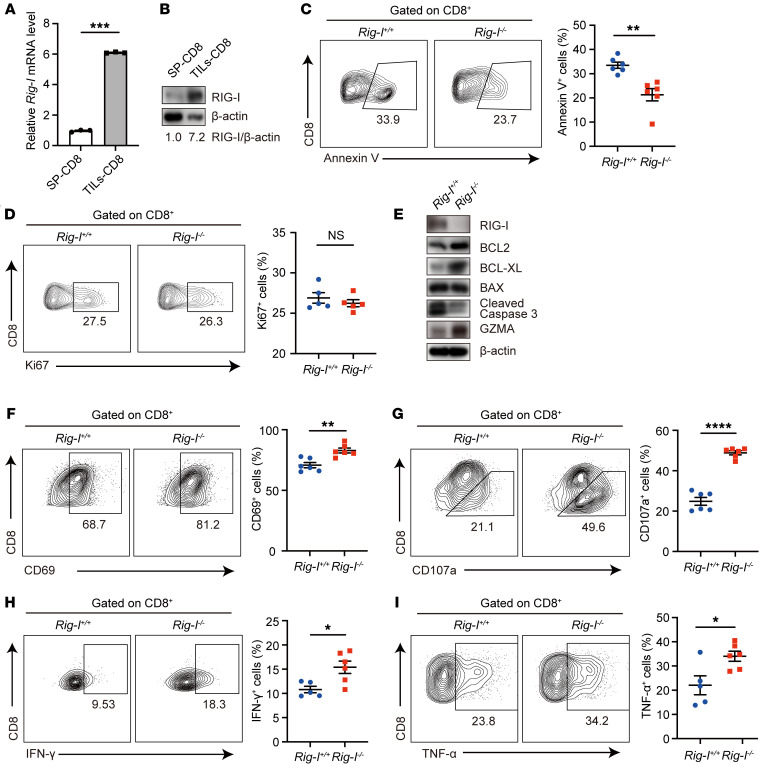
*Rig-I* ablation strengthens survival and antitumor capacity of tumor-infiltrating CD8^+^ T cells. (**A** and **B**) qRT-PCR analysis of *Rig-I* mRNA level (**A**) and Western blotting analysis of RIG-I protein level (**B**) in CD45^+^CD8^+^ cells sorted from spleens or MC38 tumor-infiltrating lymphocytes of WT mice. (**C** and **D**) The percentages of Annexin V^+^ cells (**C**) or Ki67^+^ cells (**D**) in CD45^+^ CD8^+^ cells from MC38 tumor-infiltrating lymphocytes in *Rig-I^+/+^* or *Rig-I^–/–^* mice were determined by flow cytometry. Representative cytometric plots and quantification plots are shown. (**E**) The protein levels of proapoptotic and antiapoptotic proteins and granzyme A were detected in freshly isolated tumor-infiltrating CD8^+^ T cells by Western blotting. The same batch of samples were run contemporaneously in different gels. (**F**–**I**) Indicated markers of MC38 tumor-infiltrating CD8^+^ T cells were determined by flow cytometry. Representative cytometric plots and quantification plots are shown. Data are representative of at least 2 independent experiments and expressed as mean ± SEM. *P < 0.05, **P < 0.01, ***P < 0.001, ****P < 0.0001, by unpaired Student’s *t* test.

Interestingly, *Rig-I* expression was significantly elevated in tumor-infiltrating CD8^+^ T cells compared with spleen CD8^+^ T cells (Figure 2, A and B). Flow cytometry and Western blotting assays revealed an enhanced survival profile of intratumoral *Rig-I^–/–^* CD8^+^ T cells compared with intratumoral *Rig-I^+/+^* CD8^+^ T cells, as evident by measurement of Annexin V, antiapoptotic proteins (BCL2 and BCL-XL), and pro-apoptotic proteins (BAX and Cleaved Caspase 3), while no obvious difference in proliferation kinetics was detected between the 2 groups of CD8^+^ T cells (Figure 2, C–E). By examining the levels of effector molecules (CD69, CD107a, GZMA, IFN-γ, TNF-α, GZMB, and Perforin-1), we also noticed that the tumor-infiltrating CD8^+^ T cell effector response was significantly enhanced by *Rig-I* deficiency (Figure 2, E–I, and Supplemental Figure 2A). Nevertheless, the expression of exhaustion-associated proteins in tumor-infiltrating CD8^+^ T cells — including immunosuppressive receptors PD-1, TIM-3, TIGIT, and LAG3 — were not affected by *Rig-I* deficiency (Supplemental Figure 2B). Correspondingly, there was also no significant difference in PD-L1 expression of tumor cells between the tumor-inoculated Rig-I^+/+^ and Rig-I^–/–^ mice (Supplemental Figure 2C). Taken together, these results suggest that the inhibited growth of several types of tumors in *Rig-I^–/–^* mice is at least partially attributed to the increased accumulation and functionality of CD8^+^ T cells.

### Rig-I deficiency promotes the survival and cytotoxicity of in vitro activated CD8^+^ T cells.

**Figure 3:**
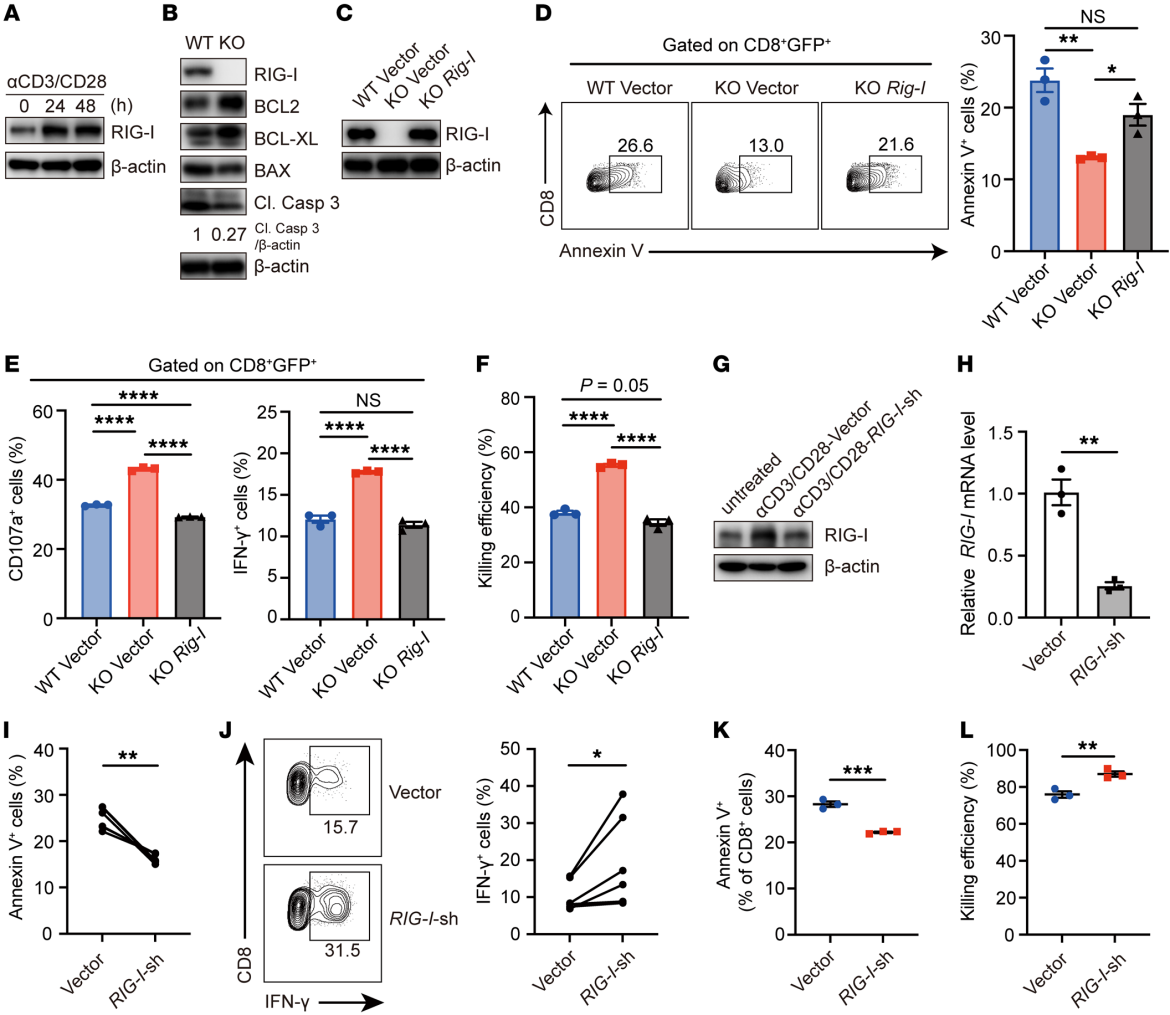
*Rig-I* ablation promotes survival and effector function of in vitro activated CD8^+^ T cells. (**A**) RIG-I protein levels in freshly isolated mouse splenic naive CD8^+^ T cells or anti-CD3/CD28–stimulated CD8^+^ T cells were determined by Western blotting. (**B**) Indicated protein levels were detected by Western blotting in *Rig-I^+/+^* (WT) or *Rig-I^–/–^* (KO) naive CD8^+^ T cells after 48 hour anti-CD3/CD28–stimulation. The same batch of samples were run contemporaneously in different gels. (**C–F**) CD8^+^ T cells were retrovirally transfected with control-GFP (vector) or *Rig-I*-GFP (*Rig-I*). RIG-I protein levels of sorted GFP^+^ cells were determined by Western blotting (**C**). The percentages of GFP^+^ cells with Annexin V^+^ staining (**D**) and CD107a^+^ or IFN-γ^+^ staining (**E**) were determined by flow cytometry. GFP^+^ cells were cocultured with MC38 cells at 10-to-1 ratio overnight and killing efficiencies were determined (**F**). (**G**–**J**) CD8^+^ T cells sorted from human PBMCs were stimulated by anti-CD3/CD28 overnight and retrovirally transfected with NC-GFP (Vector) or *RIG-I*-shRNA-GFP (*RIG-I*-sh). RIG-I protein levels of unstimulated CD8^+^ T cells and sorted GFP^+^ cells were detected by Western blotting (**G**) and *RIG-I* mRNA levels of sorted GFP^+^ cells were detected by qRT-PCR (**H**). Annexin V (**I**) and IFN-γ (**J**) expression were analyzed by flow cytometry. (**K** and **L**) CD19-CAR-T cells were retrovirally infected with NC-GFP (Vector) or *RIG-I*-shRNA-GFP (*RIG-I*-sh) in vitro. The proportion of Annexin V^+^ cells in CD8^+^GFP^+^ cells was detected by flow cytometry (**K**). Sorted GFP^+^ cells were cocultured with Nalm6 cells at the ratio of 1-to-2 overnight, and killing efficiency was determined (**L**). Data are representative of 3 independent experiments and expressed as mean ± SEM. *P < 0.05, **P < 0.01, ****P < 0.0001, by 1-way ANOVA (**D**–**F**), unpaired Student’s *t* test (**H**, **K**, and **L**) or paired Student’s *t* test (**I** and **J**).

To test a possible direct regulatory effect of cellular intrinsic RIG-I onto CD8^+^ T cells, we sorted out naive CD8^+^ T cells from the spleens of WT or *Rig-I^–/–^* mice and stimulated them with plates coated with CD3/CD28 antibodies. Consistent with the aforementioned observations on tumor-infiltrating CD8^+^ T cells (Figure 2B), RIG-I protein levels increased with CD8^+^ T cell activation (Figure 3A). Moreover, activated *Rig-I^–/–^* CD8^+^ T cells expressed higher levels of antiapoptotic proteins and lower levels of proapoptotic proteins than WT CD8^+^ T cells (Figure 3B). Additionally, significantly decreased levels of apoptosis of *Rig-I^–/–^* CD8^+^ T cells compared with WT CD8^+^ T cells, indicated by Annexin V measurement, was observed after 2 days of stimulation, which was rectified by retrovirally reintroducing a WT *Rig-I* expressional cassette into *Rig-I^–/–^* CD8^+^ T cells (Figure 3, C and D). Additionally, *Rig-I^–/–^* CD8^+^ T cells expressed significantly higher levels of markers associated with effector functions (CD107a and IFN-γ) than WT CD8^+^ T cells, which was again reversed by expression of exogenous RIG-I protein (Figure 3E). As expected, retroviral reintroduction of RIG-I counteracted the enhanced cytotoxicity of *Rig-I*^–/–^ CD8^+^ T cells toward MC38 tumor cells in vitro (Figure 3F). Taken together, these results demonstrate a critical role of intrinsic RIG-I in negative modulation of CD8^+^ T cell activation.

To explore the potential human CD8^+^ T cell relevance of this regulatory pathway, we activated CD8^+^ T cells isolated from peripheral blood mononuclear cells (PBMCs) of healthy donors and retrovirally transfected them with vector or *RIG-I*-shRNA. Similarly, *RIG-I* expression was induced with CD8^+^ T cell activation, while survival and IFN-γ level were increased in human CD8^+^ T cells by *RIG-I* knockdown (Figure 3, G–J). Next, we knocked down *RIG-I* in CD19-CAR-T cells in vitro, and then cocultured these CAR-T cells with Nalm6, a human acute lymphoblastic leukemia cell expressing high levels of CD19. Flow cytometry results showed that the proportion of apoptotic CAR-T cells was significantly reduced after *RIG-I* knockdown (Figure 3K), and, consistently, the killing efficiency toward target cells was significantly increased (Figure 3L). These results indicate that RIG-I in activated human CD8^+^ T cells impedes both their survival and cytotoxicity.

### Adoptively transferred Rig-I–deficient CD8^+^ T cells exhibit potent antitumor activity.

**Figure 4:**
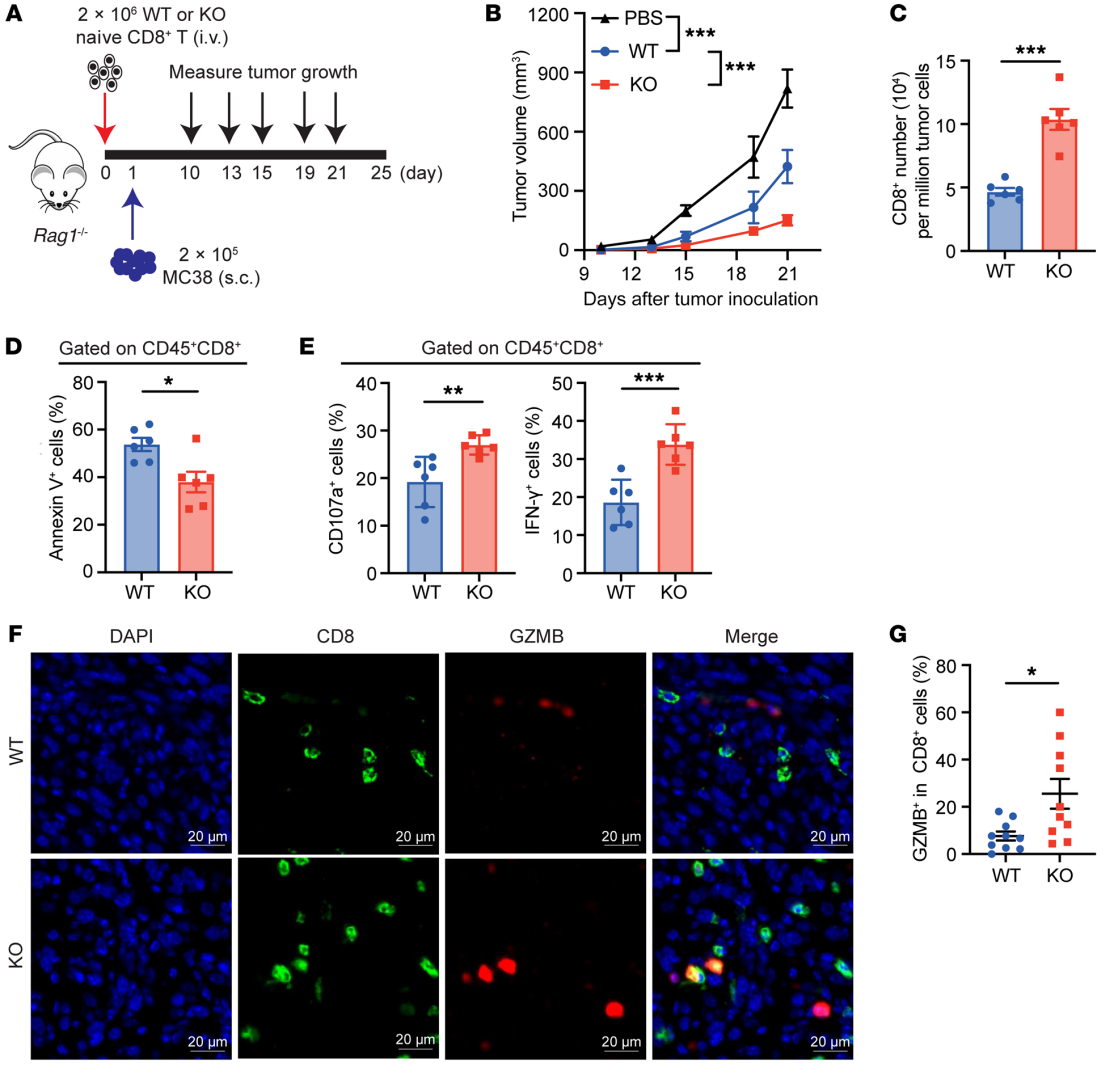
Intrinsic *Rig-I* deficiency boosts antitumor activity of adoptively transferred CD8^+^ T cells. (**A** and **B**) 2 × 10^6^ Naive CD8^+^ T cells from spleens of *Rig-I^+/+^* or *Rig-I^–/–^* mice were i.v. transfused into *Rag1^–/–^* mice. Saline was used as a vehicle control. 2 × 10^5^ MC38 cells were s.c. inoculated 1 day after T cell injection and tumor volume was measured on days 10, 13, 15, 19, and 21 (*n* = 6 per group). Experimental design (**A**) and tumor growth curve (**B**) are shown. (**C**) CD45^+^CD8^+^ cell number was counted using flow cytometry. (**D** and **E**) Annexin V, IFN-γ, and CD107a were detected by flow cytometry. The percentage of Annexin V^+^ cells (**D**) and IFN-γ^+^ or CD107a^+^ cells (**E**) in CD45^+^CD8^+^ cells are shown. (**F** and **G**) Immunofluorescence staining of tumor tissue was performed to detect the nuclei (DAPI; blue), CD8 (green) and granzyme B (red). 10 random fields of view for each group were analyzed. Representative images (**F**) and combined plots (**G**) of the percentages of GZMB^+^CD8^+^ T cells were shown. Data are representative of 2 independent experiments and expressed as mean ± SEM. *P < 0.05, **P < 0.01, ***P < 0.001, by 2-way ANOVA (**B**) or unpaired Student’s *t* test (**C**–**E** and **G**).

Next, to address whether naïve *Rig-I*^–/–^ CD8^+^ T cells behave similarly within the TME in vivo, we performed an adoptive T cell transfer experiment into tumor-bearing syngeneic recipients. We isolated naive CD8^+^ T cells from the spleens of *Rig-I^+/+^* or *Rig-I^–/–^* mice and i.v. transfused them into *Rag1^–/–^* immunodeficient mice. After 1 day, MC38 tumor cells were subcutaneously inoculated and tumor growth was monitored (Figure 4A). As measured in the spleens 25 days after i.v. infusion, both *Rig-I^+/+^* and *Rig-I^–/–^* CD8^+^ T cells were successfully engrafted in *Rag1^–/–^* mice (Supplemental Figure 3A). The infused *Rig-I^–/–^* naive CD8^+^ T cells resulted in stronger tumor growth–inhibitory effect than the infused *Rig-I^+/+^* naive CD8^+^ T cells (Figure 4B). Moreover, flow cytometric analysis and IHC showed increased infiltration of *Rig-I^–/–^* CD8^+^ T cells compared with *Rig-I^+/+^* CD8^+^ T cells in the tumor (Figure 4C and Supplemental Figure 3B); as expected, the tumor-infiltrating *Rig-I^–/–^* CD8^+^ T cells exhibited less cell death but increased IFN-γ and CD107a levels than the tumor-infiltrating WT CD8^+^ T cells (Figure 4, D and E, and Supplemental Figure 3C). The elevated expression of GZMB in tumor-infiltrating *Rig-I^–/–^* CD8^+^ T cells was verified by immunofluorescence (Figure 4, F and G), and Supplemental Figure 3D). Likewise, the growth of poorly immunogenic B16F10 cells s.c. inoculated into *Rag1^–/–^* mice was also significantly delayed by *Rig-I^–/–^* naive CD8^+^ T cells compared with saline control and *Rig-I^+/+^* naive CD8^+^ T cells (Supplemental Figure 3, E–G). Increased infiltration of *Rig-I^–/–^* CD8^+^ T cells compared with *Rig-I^+/+^* CD8^+^ T cells was also found in inoculated B16F10 tumors (Supplemental Figure 3H), and less cell death but increased IFN-γ levels of tumor-infiltrating *Rig-I^–/–^* CD8^+^ T cells than WT CD8^+^ T cells were detected by flow cytometry (Supplemental Figure 3, I and J).

**Figure 5:**
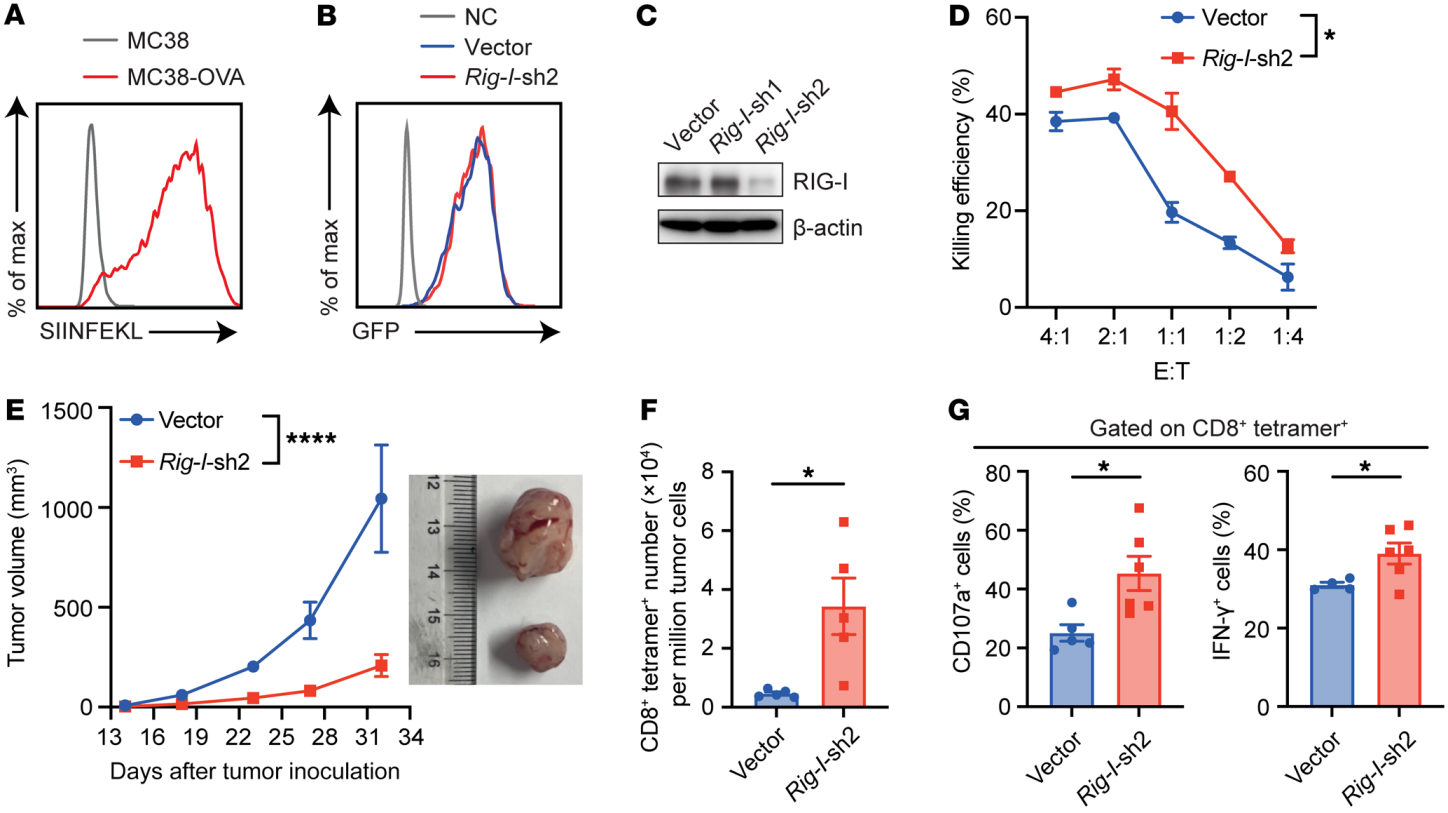
*Rig-I* deficiency enhances antitumor activity of transferred antigen-specific CD8^+^ T cells. (**A**) The presentation of OVA-derived SIINFEKL peptide by H-2Kb in MC38 or puromycin-screened MC38-OVA cells was analyzed by flow cytometry. (**B** and **C**) Splenic CD8^+^ T cells from OT-I mice were retrovirally transfected with control-GFP (vector) or *Rig-I*-shRNA-GFP (*Rig-I*-shRNA). The expression of GFP was confirmed by flow cytometry (**B**) and RIG-I protein level of GFP^+^ cells was analyzed by Western blotting (**C**). (**D**) Vector or *Rig-I*-shRNA-2–transfected CD8^+^ T cells from spleens of OT-I mice were cocultured with MC38-OVA cells at indicated E-to-T ratios and killing efficiency was analyzed by flow cytometry. (**E**) 2 × 10^5^ MC38-OVA tumor cells were s.c. inoculated in WT C57 mice. CD8^+^ T cells isolated from the spleens of OT-I mice were retrovirally transfected with vector or *Rig-I*-shRNA-2, and a total of 2 × 10^6^ infected OT-I cells were transferred into mice bearing MC38-OVA tumor (*n* = 5 per group) when the tumor was visible. Tumor growth curve and representative picture of tumors retrieved from mice on day 34 are shown. (**F** and **G**) Tumors were extracted 34 days later after tumor inoculation for tumor-infiltrating CD8^+^tetramer^+^ T cell analysis. CD8^+^tetramer^+^ T cell number was counted (**F**) and the percentages of IFN-γ^+^ or CD107a^+^ cells (**G**) of antigen-specific CD8^+^ T cells were analyzed by flow cytometry. Data are representative of 2 independent experiments and expressed as mean ± SEM. *P < 0.05, ****P < 0.0001, by 2-way ANOVA (**D** and **E**) or unpaired Student’s *t* test (**F** and **G**).

We then utilized the OT-I mouse model (33) to test the possible effect of RIG-I on antigen-specific CD8^+^ T cells. After infection with ovalbumin-expressing (OVA-expressing) retrovirus, the MC38-OVA cell line was established by puromycin selection (Figure 5A). Similarly, through infecting CD8^+^ T cells isolated from spleens of OT-I mice with *Rig-I*-shRNA-GFP–expressing retrovirus, we obtained the sorted GFP^+^ CD8^+^ T cells that exhibited diminished RIG-I protein level (Figure 5, B and C). Of note, *Rig-I* knockdown in antigen-specific CD8^+^ T cells rendered more cell death of cocultured MC38-OVA tumor cells at distinct E-to-T ratios in vitro (Figure 5D). We next transferred OT-I CD8^+^ T cells into WT mice implanted with MC38-OVA cells (Supplemental Figure 4A). Consistent with the results mentioned above (Figure 4B), *Rig-I* knockdown greatly enhanced the capacity of OT-I CD8^+^ T cells to restrict MC38-OVA tumor growth (Figure 5E), and this phenomenon was accompanied with greatly increased accumulation of the antigen-specific CD8^+^ T cell (Figure 5F) and elevated expression of CD107a and IFN-γ (Figure 5G). After confirming the expression of OVA in B16-OVA cell line (Supplemental Figure 4B), antigen-specific CD8^+^ T cells with normal or diminished RIG-I expression were cocultured with B16-OVA tumor cells in vitro*,* which showed that *Rig-I* knockdown significantly elevated the killing efficiency of antigen-specific CD8^+^ T cells (Supplemental Figure 4C). Consistently, *Rig-I* knockdown greatly enhanced the capacity of OT-I CD8^+^ T cells to restrict B16-OVA tumor growth (Supplemental Figure 4D), and antigen-specific CD8^+^ T cell infiltration (Supplemental Figure 4E) and higher levels of CD107a and IFN-γ expression (Supplemental Figure 4F) were also detected. Taken together, these results indicate that *Rig-I* deficiency or inhibition in CD8^+^ T cells might be used as a general strategy to boost antitumor immunity of adoptively transferred T cells.

### Rig-I ablation broadly reprograms the expressional profiles of activated CD8^+^ T cells independently of the classic RLR signaling pathway.

**Figure 6:**
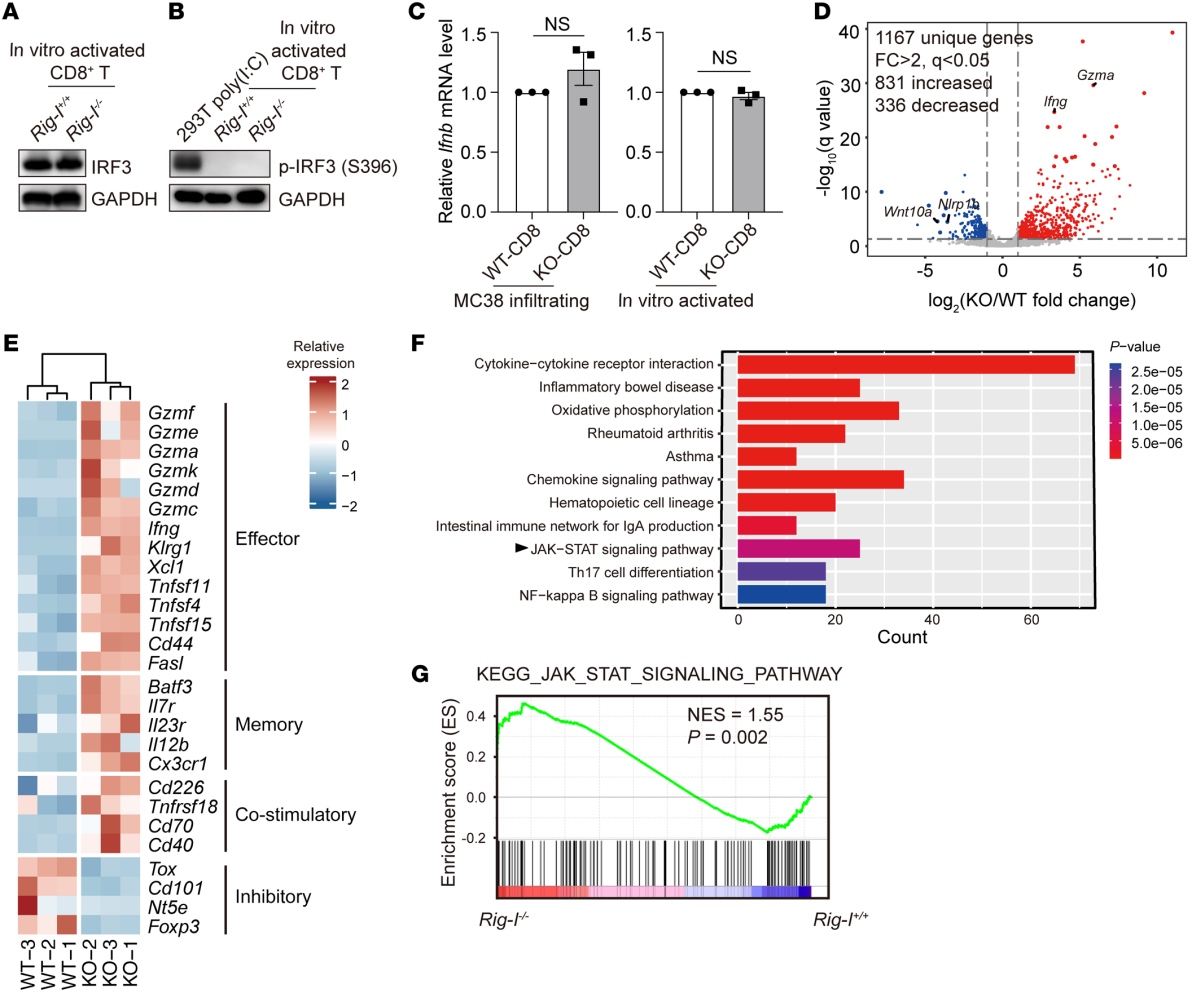
*Rig-I* ablation broadly reprograms the expression profiles of activated CD8^+^ T cells independently of the classic RLR pathway. (**A** and **B**) Naive CD8^+^ T cells from spleens of *Rig-I^+/+^* or *Rig-I^–/–^* mice were stimulated by plate-coated anti-CD3/CD28 for 48 hours and indicated protein levels were analyzed by Western blotting. 293T cells transfected with 100 ng poly (I:C) were used as positive control for p-IRF3 (S396) detection. (**C**) *Ifnb* relative mRNA levels were measured in naive CD8^+^ T cells stimulated by anti-CD3/CD28 for 48 hours or tumor-infiltrating CD8^+^ T cells of MC38 inoculated *Rig-I^+/+^* or *Rig-I*^–/–^ mice. (**D**–**G**) Naive *Rig-I^+/+^* or *Rig-I^–/–^* CD8^+^ T cells were cultured under the stimulation of anti-CD3/CD28 for 48 hours and submitted for RNA-Seq. Scatter plot comparing global gene-expression profiles of in vitro activated *Rig-I^+/+^* or *Rig-I^–/–^* CD8^+^ T cells (**D**). Genes with FC > 2 and *q* < 0.05 are shown in red (increased expression; 831 genes) or blue (decreased expression; 336 genes). Heat map showing indicated gene expression between 2 groups (**E**). Histogram demonstrating the KEGG analysis for the signaling pathways that were significantly different between in vitro activated *Rig-I^+/+^* and *Rig-I^–/–^* CD8^+^ T cells (**F**). GSEA showing enriched JAK-STAT signaling in *Rig-I^–/–^* CD8^+^ T cells compared with WT counterparts (**G**). Data are representative of 3 independent experiments (**A**–**C**) and expressed as mean ± SEM (**C**).

We wondered whether the status of the classic RLR pathway would be altered by *Rig-I* deficiency in activated CD8^+^ T cells in vitro. Once activated by RNA ligands, RIG-I triggers the assembly of the MAVS that signal for phosphorylation of IRF3 (34), which was undetectable in the in vitro activated *Rig-I^+/+^* or *Rig-I^–/–^* CD8^+^ T cells (Figure 6, A and B). In addition, *Ifnb* mRNA levels were similarly presented in either tumor-infiltrating Rig-I^+/+^ and Rig-I^–/–^ CD8^+^ T cells or in vitro-activated Rig-I^+/+^ and Rig-I^–/–^ CD8^+^ T cells (Figure 6C). Therefore, the enhanced survival or cytotoxicity of Rig-I^–/–^ CD8^+^ T cells was not likely due to any alteration of the RLR axis.

On the other hand, as evidenced by principal component analysis (PCA) (Supplemental Figure 5A) of RNA-Seq data of the in vitro stimulated naive *Rig-I^+/+^* or *Rig-I^–/–^* CD8^+^ T cells, *Rig-I* deficiency caused broad alterations in the expression profile (Supplemental Table 1). As displayed in the volcano plot and heatmap, we found a dramatic shift in the expression of many genes in CD8^+^ T cells in response to *Rig-I* depletion (Figure 6D and Supplemental Figure 5B). It is worth noting that *Rig-I* deficiency rendered upregulated expression of multiple effector-related genes and some genes facilitating T cell memory and costimulation, while some inhibitory genes were downregulated by *Rig-I* deficiency (Figure 6, D and E). KEGG analysis suggested that more pathways involving oxidative phosphorylation, cytokine production, and JAK-STAT signaling were among top-ranked differentially expressed pathways (Figure 6F). Gene Ontology (GO) enrichment analysis indicated that genes involved in mitochondrial membrane and cytokine production were significantly changed (Supplemental Figure 5, C and D). In accordance, gene set enrichment analysis (GSEA) highlighted a relevance of *Rig-I* deficiency to JAK-STAT signaling (Figure 6G) and oxidative phosphorylation pathways (Supplemental Figure 5E). Taken together, these data show that *Rig-I* deficiency greatly alters expression profiles of activated CD8^+^ T cells, implicating JAK-STAT pathway as one of the candidate nodal pathways potentially related to the enhanced survival and cytotoxicity of the in vitro activated *Rig-I^–/–^* CD8^+^ T cells.

### RIG-I restrains CD8^+^ T cell survival and cytotoxicity by dampening the HSP90-mediated protective effect on STAT5 phosphorylation.

**Figure 7:**
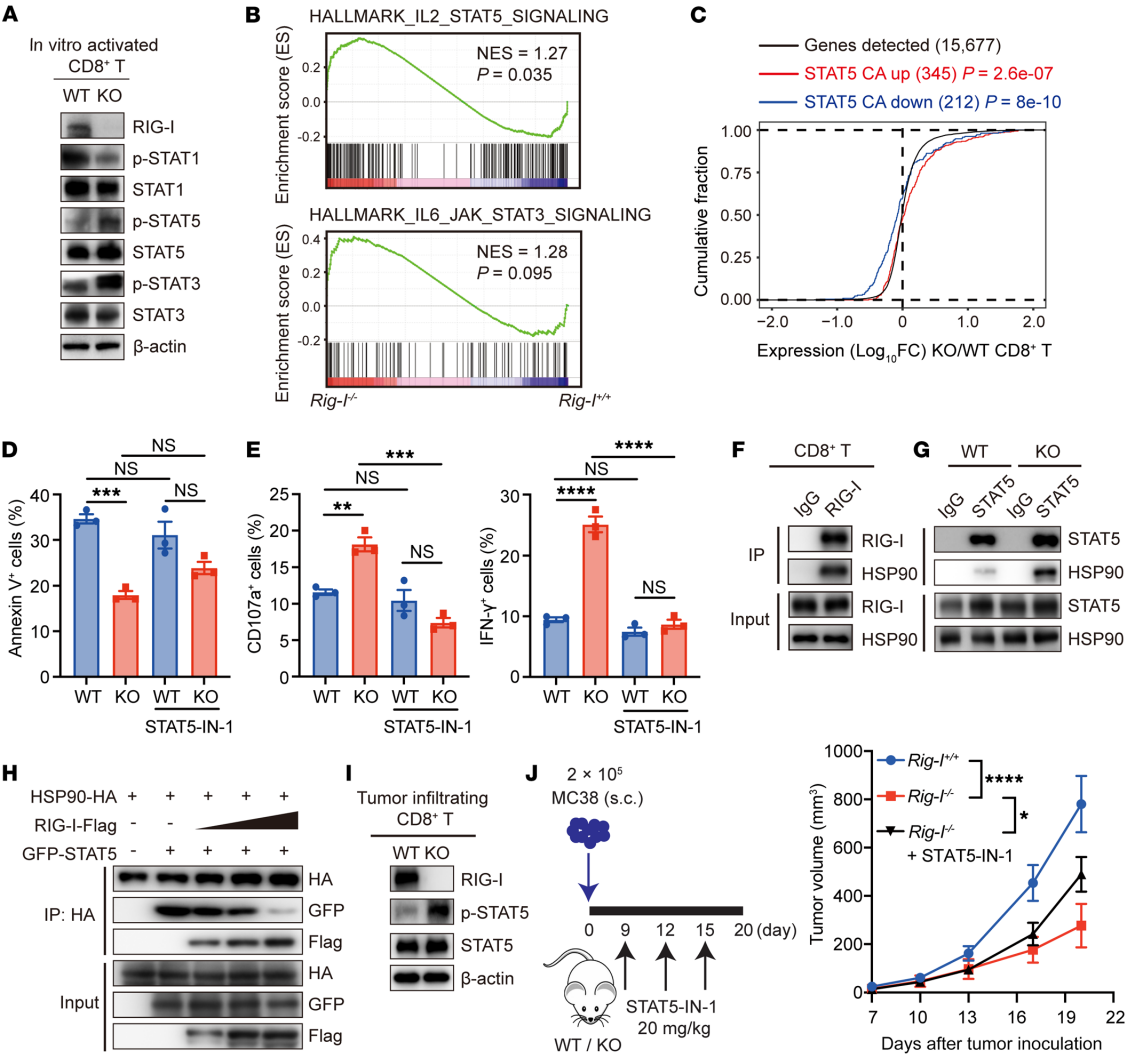
RIG-I restrains CD8^+^ T cell survival and cytotoxicity by dampening HSP90-mediated protective effect on STAT5. (**A**) Indicated protein levels of splenic naive CD8^+^ T cells after 48 hours anti-CD3/CD28 stimulation were analyzed by Western blotting. (**B**) GSEA of RNA-Seq (same as Figure 6, D–G) comparing in vitro activated CD8^+^ T cells as indicated are shown. (**C**) Empirical cumulative distribution function for change in expression of all genes (black) expressed in in vitro activated *Rig-I^–/–^* CD8^+^ T cells (change relative to that in *Rig-I^+/+^* CD8^+^ T cells) and for subsets of genes upregulated (red) or downregulated (blue) by overexpression of constitutively active STAT5 in CD8^+^ T cells. (**D** and **E**) Splenic naive CD8^+^ T cells were stimulated by anti-CD3/CD28 and STAT5-IN-1 (10 μM) for 2 days and analyzed by flow cytometry. (**F** and **G**) Cell lysates of in vitro stimulated CD8^+^ T cells were immunoprecipitated with dynabeads-coupled control IgG or RIG-I antibody (**F**). Cell lysates of naive *Rig-I^+/+^* or *Rig-I^–/–^* CD8^+^ T cells stimulated with anti-CD3/CD28 for 2 days were immunoprecipitated with dynabeads-coupled control IgG or STAT5 antibody (**G**). The precipitates were then probed with indicated antibodies. (**H**) Lysates of 293T cells cotransfected with indicated plasmids were immunoprecipitated with HA antibody and analyzed by immunoblot with indicated antibodies. (**I**) Indicated protein levels of MC38 tumor-infiltrating CD45^+^CD8^+^ T cells were analyzed by Western blotting. (**J**) Mice s.c. inoculated with MC38 cells were treated with STAT5-IN-1. Schematic diagram and tumor growth curve are shown (*n* = 5 per group). Data are representative of at least 2 independent experiments and expressed as mean ± SEM. *P < 0.05, **P < 0.01, ***P < 0.001, ****P < 0.0001, by 1-way ANOVA (**D** and **E**) or 2-way ANOVA (**J**). The same batch of samples were run contemporaneously in different gels for **A** and **I**.

Interestingly, although the phosphorylation levels of both STAT5 and STAT3 (but not STAT1) were elevated in *Rig-I^–/–^* CD8^+^ T cells compared with WT CD8^+^ T cells upon in vitro activation (Figure 7A), GSEA indicated that IL-2-STAT5 signaling, rather than IL-6-STAT3 signaling, was most likely responsible for *Rig-I* deficiency-caused alterations (Figure 7B). In accordance, gene-expression changes due to *Rig-I* deficiency were positively correlated with those in the constitutively activated STAT5 mutant STAT5 H298R/S710F–overexpressing CD8^+^ T cells (35) (Figure 7C). SH-4-54 (a STAT3/5 dual inhibitor) or STAT5-IN-1 (a STAT5 inhibitor) rather than stattic (a STAT3 inhibitor) (Supplemental Figure 6A) restrained survival and decreased IFN-γ and CD107a levels in *Rig-I^–/–^* CD8^+^ T cells (Figure 7, D and E, and Supplemental Figure 6, B and C). In line with this, knockdown of *Stat5* (Supplemental Figure 6D) in *Rig-I^–/–^* CD8^+^ T cells decreased survival and expression of CD107a and IFN-γ in *Rig-I^–/–^* CD8^+^ T cells, which was accompanied by compromised cytotoxicity toward MC38 cells in vitro (Supplemental Figure 6, E–G).

To comprehend the direct molecular mechanism underlying the RIG-I regulatory effect on STAT5 in activated CD8^+^ T cells, we subjected RIG-I-Flag-transfected 293T lysates to immunoprecipitation using anti-Flag magnetic beads or beads coupled with control IgG (Supplemental Figure 7A). Mass spectrometric analyses of the immunoprecipitated proteins identified multiple species that specifically associated with RIG-I (Supplemental Table 2). Among those that physically partnered with RIG-I, molecular chaperone HSP90 caught our attention because it was shown to facilitate and prolong JAK-STAT signaling activation by changing the STAT conformation to maintain its phosphorylation (36–38). RIG-I-HSP90 association has been reported by others (39) and verified by our own coimmunopreciptiated(co-IP) data in 293T cells (Supplemental Figure 7B). Indeed, co-IP assay demonstrated that endogenous RIG-I/HSP90 association existed in in vitro-activated CD8^+^ T cells (Figure 7F). Interestingly, the association of HSP90/STAT5 was increased by *Rig-I* deficiency (Figure 7G), which prompted us to hypothesize that RIG-I induction upon CD8^+^ T cell activation might sequester HSP90 from associating with and promoting STAT5 activation. In corroboration with this, RIG-I overexpression inhibited HSP90/STAT5 association in a dose-dependent manner (Figure 7H). As revealed by domain-mapping experiments performed by Matsumiya et al. (39), helicase and C-terminal domains of RIG-I were responsible for the association with HSP90. On the other hand, HSP90 middle and C-terminal domains were responsible for the association with RIG-I (Supplemental Figure 7C), suggesting that the homo-dimer structure of HSP90 may be crucial for its interaction with RIG-I (40).

Expectedly, we detected an elevated STAT5 activation (p-STAT5 [Tyr694]) level in freshly isolated MC38 tumor-infiltrating *Rig-I^–/–^* CD8^+^ T cells compared with their WT counterparts (Figure 7I). Immunofluorescence showed that more STAT5 protein colocalized with HSP90 in the presence of RIG-I deficiency (Supplemental Figure 7D). Ex vivo inhibition of HSP90 or STAT5 at least partially decreased survival and effector function of tumor-infiltrating *Rig-I^–/–^* CD8^+^ T cells (Supplemental Figure 7, E–H). These results indicate that RIG-I restrains STAT5 phosphorylation within tumor-infiltrating CD8^+^ T cells via HSP90. Consistently, SH-4-54 or STAT5-IN-1 treatment in vivo rescued tumor growth inoculated in *Rig-I^–/–^* mice (Figure 7J and Supplemental Figure 7, I–K), thus implicating a functional contribution of STAT5 overactivation to the enhanced antitumor effect of *Rig-I^–/–^* CD8^+^ T cells. The p-STAT5 (Tyr694) level was also elevated in human CD8^+^ T cells transfected with *RIG-I*-shRNA compared with the control group (Supplemental Figure 7L), indicating that RIG-I also restrains STAT5 overactivation in activated human CD8^+^ T cells.

### Frequency of RIG-I^+^ tumor-infiltrating CD8^+^ T cells associates with the poor prognosis of patients with colon cancer.

**Figure 8:**
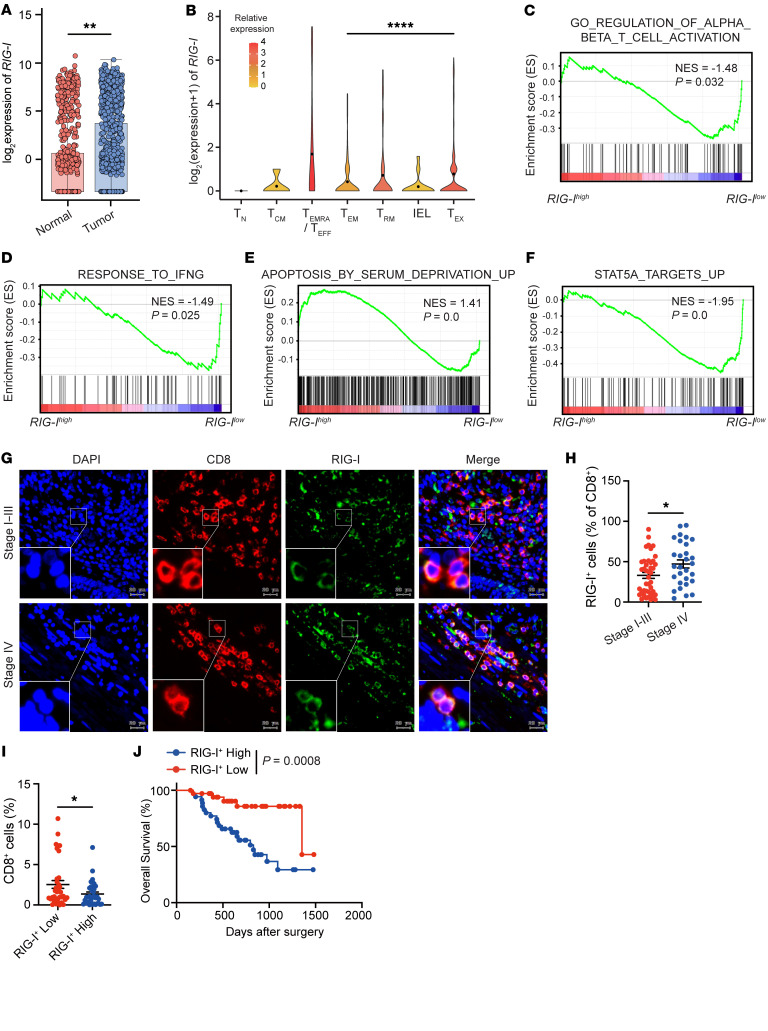
Frequency of RIG-I^+^ tumor-infiltrating CD8^+^ T cells associates with the poor prognosis for patients with colon cancer. (**A**–**F**) Publicly available scRNA-Seq data from 12 individuals with CRC were analyzed, focusing on tumor-infiltrating CD8^+^ T cells and CD8^+^ T cells from adjacent nonmalignant tissues. Data are from GSE108989. Box plot showing the expression of *RIG-I* in CD8^+^ T cells from adjacent nonmalignant tissues (Normal) or tumor tissues (Tumor) (**A**). Violin plot showing the expression of *RIG-I* in indicated subsets of CD8^+^ T cells (**B**). GSEA plots showing enriched T cell activation (**C**), IFN-γ response (**D**), apoptosis by serum deprivation (**E**) and STAT5a target gene signatures (**F**) in tumor-infiltrating CD8^+^ T cells with high versus low *RIG-I* expression. (**G** and **H**) Slides of tumor tissue from colorectal carcinoma patients were costained by RIG-I antibody (green) and CD8 antibody (red). DAPI was used to visualize the nuclei (blue). Representative images (**G**) and the proportion of CD8^+^RIG-I^+^ cells of CD8^+^ cells from stage I–III or stage IV patients were shown (**H**). (**I**) The percentages of CD8^+^ T cells in nucleated cells for RIG-I^+^ CD8^+^ low group or RIG-I^+^ CD8^+^ high group are shown. (**J**) Kaplan-Meier plot depicting OS of patients from RIG-I^+^CD8^+^–low group or RIG-I^+^CD8^+^–high group are shown. Data are expressed as mean ± SEM. *P < 0.05, **P < 0.01, ****P < 0.0001, by unpaired Student’s *t* test (**A**, **B**, **H**, and **I**) or log-rank test (**J**).

To examine a possible clinical relevance of our findings, we reanalyzed publicly available single cell RNA–Seq data from 12 individuals with colorectal cancer (41) by focusing on tumor-infiltrating CD8^+^ T cells from tumor tissues paired with CD8^+^ T cells in adjacent nonmalignant tissues. Tumor-infiltrating CD8^+^ T cells had a significantly higher mRNA level of *RIG-I* than adjacent nonmalignant tissues (Figure 8A). This result was validated by cell type–level expression analysis from GEPIA 2021 (http://gepia2021.cancer-pku.cn) comparing *RIG-I* expression between tumor-infiltrating CD8^+^ T cells and CD8^+^ T cells in the relevant normal tissues (Supplemental Figure 8A). Consistent with previous data showing that activated CD8^+^ T cells expressed higher levels of RIG-I (Figure 3A), naive and central memory had the lowest *RIG-I* expression among the various subsets of CD8^+^ T cells, while recently activated effector memory or effector T cells showed the highest levels of *RIG-I* expression (Figure 8B). Effector memory CD8^+^ T cells expressed a significantly lower level of *RIG-I* compared with exhausted CD8^+^ T cells (Figure 8B), implying that CD8^+^ T cells with lower expression of *RIG-I* may have better cytotoxic potential. Further analysis of CD8^+^ T cells from tumor tissues demonstrated the enrichment of IFN-γ response and T cell activation gene signature pathways in tumor-infiltrating CD8^+^ T cells with lower *RIG-I* expression compared with tumor-infiltrating CD8^+^ T cells with higher *RIG-I* expression (Figure 8, C and D). In contrast, the apoptotic gene signature was enriched in CD8^+^ T cells with higher *RIG-I* expression (Figure 8E). Moreover, GSEA showed the enrichment of STAT5 target genes in tumor-infiltrating CD8^+^ T cells displaying lower *RIG-I* expression (Figure 8F). Therefore, RIG-I may negate human CD8^+^ T cell survival and functional potency via the STAT5 signaling pathway in human tumor tissues.

To further explore the relationship between RIG-I expression in tumor-infiltrating CD8^+^ T cells and the prognosis of tumor patients, we collected tumor tissues of patients with colorectal cancer (CRC) whose clinicopathological features were detailed in Table 1. These patients all underwent surgical resection of the primary tumors, and both overall survival (OS) and progression-free survival (PFS) were monitored. Through performing coimmunofluorescence staining to detect RIG-I and CD8, we found that the percentage of double-positive (RIG-I^+^ CD8^+^) cells in CD8^+^ cells was significantly higher in patients in stage IV compared with those in patients in stages I–III (Figure 8, G and H). Patients with higher RIG-I^+^CD8^+^ T cell rate had less CD8^+^ T cell infiltration in tumor tissues (Figure 8I). The expression of RIG-I in CD8^+^ T cells significantly correlated with worse OS prognosis of patients with CRC (Figure 8J and Supplemental Figure 8B), which was mostly caused by the difference occurring to the stage IV patients (Supplemental Figure 8C). Furthermore, clinicopathological factors were integrated into multivariate analysis, which pointed to the percentage of RIG-I^+^ in CD8^+^ cells, along with lympho-vascular invasion, as an independent predictor of OS (Table 1). On the other hand, although PD-1 and TIM-3 expression increased with human CD8^+^ T cell activation, *RIG-I* deficiency reduced TIM-3 elevation (Supplemental Figure 8D); these inhibitory markers seemed not to change when RIG-I expression varied in tumor-infiltrating CD8^+^ T cells of tumor samples (Supplemental Figure 8, E and F). Taken together, these observations suggest that RIG-I induction in tumor-infiltrating CD8^+^ T cells of patients with advanced colorectal cancer may promote tumor progression, since elevated RIG-I expression should compromise CD8^+^ T cells’ survival and cytotoxicity.

## Discussion

Using *Rig-I^–/–^* tumor–bearing mouse models, we were the first to demonstrate that intrinsic RIG-I antagonizes CD8^+^ T cell survival and effector function in the TME and undermines CD8^+^ T cell-mediated antitumor immunity (Figures 1 and 2). This conclusion was supported by data generated in in vitro activated mouse CD8^+^ T cells (Figure 3) and complemented by studies in tumor-bearing mouse models injected with adoptively transferred CD8^+^ T cells with normal or deficient *Rig-I* expression (Figures 4 and 5). As an extension of this research, data mined in public databases and our own analysis on primary colorectal cancer samples revealed a negative correlation between *RIG-I* expression in tumor-infiltrating CD8^+^ T cells and tumor progression (Figure 8). It is worth noting that the expression of RIG-I in tumor-infiltrating CD8^+^ T cells may be used to predict the prognosis of patients with colorectal cancer at the advanced stage. Therefore, we filled a knowledge gap about RIG-I regarding the functional importance of RIG-I in CD8^+^ T cell antitumor immunity and we provided evidence that RIG-I may be another immune checkpoint factor for tumors to take advantage of to evade CD8^+^ T cell-mediated immune surveillance. Certainly, further studies are needed to test these hypotheses, and, hopefully, we can find an effective way to rescue RIG-I-induction–mediated CD8^+^ T incompetence.

Extensive experimental studies augmenting tumor immune rejection via modulating RIG-I have been reported, most of which were centered around RIG-I’s role as an RNA ligand sensor (14–20). Generally, RIG-I acts as a tumor suppressor in various types of tumors, as its activation by RNA agonist within tumor cells promotes tumor cell death and increases tumor immunogenicity. Likewise, induction and activation of RIG-I within stromal or even antigen-presenting cells (APCs) activates innate immunity through classical RIG-I signaling pathways (16). Consequently, tumor antigen-specific CD8^+^ T cells usually expand and accumulate in tumor tissues, thereby synergizing with immune checkpoint blockade to inhibit tumor growth (17, 18, 20). Consistently, reexpression of the MAVS stimulates broad cellular interferon-related responses in CRCs that provoke tumor antigen–specific adaptive immunity and synergize with anti–PD-L1 (42). However, we have previously shown that RIG-I exerts a physiological role to maintain Treg/Th17 cell balance, at least in part by restraining STAT3 activation (31). Combined with our findings that the absence of *Rig-I* in CD8^+^ T cells slows tumor progression in different settings of mouse models through facilitating the interaction of HSP90 and STAT5, the activation or upregulation of RIG-I in tumor-infiltrating T cells may represent a previously unappreciated obstacle that might counteract the antitumor effects initiated from RIG-I agonists within tumor or stromal cells.

Adoptive T cell therapies have generated impressive outcomes in limited types of tumors including hematological malignancies (43) and melanoma (44). Although the presence of a series of immune cells with immunosuppressive function, including regulatory T cells, tumor-associated macrophages, myeloid-derived suppressor cells, and tumor-associated neutrophils, represent critical barriers that impede proper functioning of adoptively transferred T cells, the lack of persistence and antitumor activity of transferred T cells themselves contributes to poor efficacy (45). This revelation motivates many efforts to improve the intrinsic functionality of transferred cells. Our work showed that the TME induces RIG-I expression in CD8^+^ T cells (Figure 2, A and B), and that highly induced RIG-I in tumor-infiltrating T cells contributes to T cell death and attenuates the antitumor effect. This observation provides potential avenues to augment the antitumor efficacy of *ex vivo* expanded or engineered T cells for adoptive cell transfer by aborting RIG-I-mediated T cell death and functional impairment.

In addition, our data indicate that RIG-I in CD8^+^ T cells prevents STAT5 hyperactivation by sequestering HSP90 (Figures 6 and 7). On the one hand, STAT5 plays a major oncogenic role in the progression of several types of cancers. Aberrant STAT5 signaling, mostly due to its constitutive activation, has been found to drive tumor survival, growth, metastasis, and resistance to anticancer therapies. STAT5 inhibitors have been used to abrogate the constitutive activation of STAT5 signaling in cancer, and tyrosine kinase inhibitors (TKIs) that target the upstream signaling molecules of STAT5 — such as JAK, FLT3, and BCR-ABL — have been shown to be effective in clinical settings (46). On the other hand, STAT5 activation has been suggested to have a general role in the maintenance and expansion of T cell subsets (47). Retroviral expression of STAT5A H298R/S710F in the in vitro activated CD8^+^ T cells led to the generation and maintenance of long-lived CD8^+^ T effector memory cells (48–50). Inhibiting STAT5 activation within tumor cells while maintaining p-STAT5 level in CD8^+^ T cells may have better antitumor efficacy. HSP90, favoring active STAT3/5 localization and interaction with multiple kinases in the cytosol, is a potential therapeutic target in various kinds of cancers (51–53). HSP90 inhibitors have been suggested to robustly decrease PD-L1 surface expression and upregulate interferon response genes, thus enhancing the T cell-mediated antitumor effect (54, 55). However, most clinical trials have yielded mixed results and frequent side effects that preclude broad utilization (38). This likely, in part, comes from the observations that HSP90 inhibitors were found to selectively induce apoptosis of activated T cells and decrease the production of IFN-γ–producing and TNF-α–producing effector cells (56). Further, HSP90 inhibitors irreversibly downregulate cell surface linage markers (CD3, CD4, and CD8), costimulatory molecules (CD28, CD40L), and α-β receptors on T lymphocytes, disrupting T cell activation, proliferation, and IFN-γ production (57). Our results provide supporting evidence that maintains that the HSP90-STAT5 axis is critical for sustaining immunosurveillance of tumor-infiltrating CD8^+^ T cells and that disrupting RIG-I-HSP90 interaction may help maintain the active status of this axis.

In summary, our study revealed a novel role of RIG-I — an important innate immune molecule — in T cell–mediated antitumor immunity and pointed out an avenue to enhance T cell cytotoxicity against tumors through manipulating the RIG-I level. By analyzing both the human CRC samples that we collected and publicly accessed data, we inferred that RIG-I expression in tumor-infiltrating CD8^+^ T cells was positively associated with poor prognosis of patients with advanced CRC. Nevertheless, further studies, especially of human tumor samples in a larger scale, are needed to elucidate a possible involvement of this regulatory axis in tumor immunosurveillance and immunotherapy in a broad manner.

## Methods

### Human samples.

Human samples were obtained from healthy donors or patients with colorectal cancer admitted to Ruijin Hospital. Human PBMCs were isolated by Ficoll-Hypaque density–gradient centrifugation. CD8^+^ T cells were negatively selected by EasySep Human CD8^+^ T Cell Isolation Kit (STEMCELL) and cultured in RPMI-1640 medium (Gibco) with 10% FBS (Thermo Fisher Scientific), 1% penicillin (Thermo Fisher Scientific), and 100 μg/mL streptomycin (Thermo Fisher Scientific) in flat-bottom plates precoated with anti-human CD3 (2 μg/mL; clone OKT3; eBioscience) and anti-human CD28 (2 μg/mL; clone 28.2; eBioscience) for 1 day before transfection with NC (Vector) or *RIG-I*-shRNA and cultured for another 2 days. The shRNA sequence used to inhibit *RIG-I* expression was AGCACTTGTGGACGCTTTAAACTCGAGTTTAAAGCGTCCACAAGTGCT. The CD8^+^ T cells were stained and analyzed by flow cytometry. Colorectal cancer tissues were sliced, fixed, and incubated with primary and then secondary antibodies. Slides were mounted with DAPI. For each patient, 1–3 slides were committed to coimmunofluorescence staining. Images were scanned and analyzed by TissueFAXS cytometry. For each protein stained, every cell had its own fluorescence value, a cut-off value was determined through forward and reverse backtracking. CD8^+^, CD8^+^ RIG-I^+^, and CD8^+^ TIM-3^+^ or CD8^+^ LAG-3^+^ cells were counted. The number of total cells counted (4–20 × 10^6^) depended on the size of the slice, and all tumor-infiltrating CD8^+^ T cells were taken into consideration. Overall survival of patients with CRC was calculated according to the Kaplan-Meier method and survival was compared among different patient groups using the log rank test. The effect of patient characteristics on OS was assessed via univariate analysis using Cox proportional risk regression analysis. Hazard ratios (HRs) estimated from the Cox proportional hazard model were reported as relative risks with corresponding 95% CI (see Table 1).

### Mice.

C57BL/6 (WT) strain mice were purchased from Beijing Vital River Laboratory Animal Technology Co. Ltd. *Rig-I^–/–^* (KO) mice were acquired through backcrossing C57BL/6 WT mice with *Rig-I^–/–^* 129 strain mice for more than 10 generations (28). *Rag-1^–/–^* mice were purchased from GemPharmatech Co. Ltd. OT-I mice were purchased from Shanghai Model Organisms Center Inc. Sex-matched mice aged 6 weeks old were used unless otherwise specified. Mice were group housed with a maximum of 5 mice per cage and maintained under specific pathogen-free conditions according to the institutional guidelines for experimental animals of Shanghai Jiao Tong University School of Medicine.

### Tumor cell lines.

MC38 colon adenocarcinoma, B16F10 melanoma, and EL4 lymphoma cell lines were purchased from iCell Bioscience Inc., Shanghai. The B16-OVA cell line was a gift from the Liufu Deng lab at Shanghai Institute of Immunology. All the cell lines were tested and determined to be mycoplasma free. MC38 and B16F10 cells were maintained in DMEM (Gibco) supplemented with 10% FBS (Gibco), 100 IU/mL penicillin (Thermo Fisher Scientific), 100 mg/mL streptomycin (Thermo Fisher Scientific) at 37°C in 5% CO_2_. EL4 cells were maintained in RPMI-1640 (Gibco) supplemented with 10% FBS, 100 IU/mL penicillin, 100 mg/mL streptomycin at 37°C in 5% CO_2_. OVA cDNA was subcloned into lentiviral plasmid coexpressing the puromycin-resistance gene to produce lentivirus. MC38 cells were infected with the lentivirus for 2 days. After infection, cells were treated with 2 μg/ml puromycin for 2 days. Cells that survived puromycin and tested positive for OVA were cultured and saved as the MC38-OVA cell line.

### Tumor models.

For tumor models, 2 × 10^5^ tumor cells were injected s.c. unless otherwise specified. Tumor growth was monitored daily and measured every 3–4 days using digital callipers. Tumor volume was calculated by multiplying the longest tumor diameter and the square of its perpendicular diameter. For depletion experiments, mice were injected intraperitoneally with 100 μg isotype control (Bio X cell; BE0085 and BE0088), anti-NK1.1 (Bio X cell; BE0036), anti-CD4 (Bio X cell; BE0003) or anti-CD8β (Bio X cell; BE0223) at indicated days. shRNA sequences used to inhibit mouse CD8^+^ T *Rig-I* expression were GTTAACCCACAGTTGATCCAAATGATACTCGAGTATCATTTGGATCAACTGTGGTTTTTTCTCGAG (*Rig-I*-sh1) and GTTAACCAAGCATTCAGAGACTATATCCTCGAGGATATAGTCTCTGAATGCTTGTTTTTTCTCGAG (*Rig-I*-sh2). For STAT5 inhibitor treatment, mice were given 3 doses of i.p. injection of SH-4-54 (10 mg/kg) or STAT5-IN-1 (20 mg/kg) or vehicle control every 3 days from when the tumor was visible.

### Flow cytometry and cell sorting.

The following antibodies were used in flow cytometric analysis: CD45 (Miltenyi, 130-110-659; BD, 567111; BD, 559864), CD45.2 (BD, 552950), CD3 (BD, 557596; BD, 553064), CD4 (BD, 562891), CD8 (BD, 552877; Miltenyi, 130-118-329), CD19 (BD, 551001), NK1.1 (Biolegend, 108708; BD, 550627), CD44 (BD, 561862), CD62L (BD, 553151), CD69 (BD, 553237), CD107a (BD, 564347), Annexin V (BD, 550474), Ki67 (BD, 558615; BD, 561283), 7-AAD (BD, 555816), IFN-γ (BD, 554411), TNF-α (Invitrogen, 12-7321-82), Granzyme B (Biolegend, 396404), Perforin-1 (Biolegend, 154304), PD-1 (BD, 744548), PD-L1 (BD, 564716), TIM3 (R&D, FAB1529A), LAG3 (BD, 562346), TIGIT (Tonbo, 50-1421), OVA257-264 (SIINFEKL) peptide (Invitrogen, 17-5743-80), T-Select H-2Kb OVA Tetramer-SIINFEKL (MBL, TS-5001-2C), Negative (SIY)-Tetramer-SITRYYGL (TS-M008-2), GFP (Biolegend, 338006), Biotin anti-human CD8 (BD, 555365), PE Streptavidin (BD, 554061), IFN-γ (Biolegend, 502540), STAT1 (pY701, BD, 562985), STAT3 (pY705, BD, 612569), STAT5 (pY694, BD, 612567), TIGIT (BD, 747846), LAG-3 (BD, 565716), TIM-3 (BD, 563422), PD-1 (BD, 564494).

To obtain single-cell suspensions, tumor tissues were cut into small pieces and digested using a mouse tumor dissociation kit (Miltenyi). Spleens were mechanically dissociated into single-cell suspensions. For the staining, single-cell suspensions were preincubated with Fc-block (CD16/32) in staining buffer (PBS with 2% FBS and 2 mM EDTA) for 10 minutes at 4˚C. Dead cells were excluded with Zombie Fixable Viability Kit (Biolegend). For detection of surface markers, cells were stained with the indicated antibodies for 30 minutes at 4˚C. For intracellular staining, cells were fixed with the Foxp3/Transcription Factor Staining Buffer Set (eBioscience) in accordance with the manufacturer’s instructions. Before intracellular staining of cytokines, cells were stimulated with Cell Stimulation Cocktail plus protein transport inhibitors (eBioscience) for 4–5 hours. To measure apoptosis, cells were stained with APC-conjugated Annexin V and 7-AAD. All of the flow cytometric analyses were performed on the LSR II Fortessa cytometer (BD Biosystems) and data were analyzed using FlowJo software. For cell sorting, samples were stained with surface antibodies and sorted on a FACSAria Fusion cell sorter (BD).

### In vitro CD8^+^ T cells activation and treatment.

Mouse naive CD8^+^ T cells were negatively selected by EasySep Mouse Naive CD8^+^ T Cell Isolation Kit (STEMCELL). Isolated naive CD8^+^ T cells were cultured in RPMI-1640 medium with 10% FBS, 1% penicillin, and 100 μg/mL streptomycin in flat-bottom plates precoated with anti-CD3 (2 μg/mL; clone 145-2C11; eBioscience) and anti-CD28 (2 μg/mL; clone 37.51; eBioscience) for 2 days, unless otherwise stated. For small molecular inhibitor treatment, Stattic (Selleck, S7024), SH-4-54 (Selleck, S7337), STAT5-IN-1 (Selleck, S6784), or 17-AAG (Selleck, S1141) was added to culture medium and cells were cultured for indicated times.

### Quantitative real-time PCR.

RNA samples were isolated using RNeasy Plus Mini Kit (QIAGEN) according to the manufacturer’s procedures. Reverse-transcription reactions were performed with ReverTra Ace qPCR RT Master Mix (Toyobo) following the standard protocol. Primers were selected from PrimerBank. Quantitative RT-PCR was performed with SYBR Green Realtime PCR Master Mix (Toyobo) in 7500 Fast Real-Time PCR System or ViiA 7 Real-Time PCR System with 384-Well Block (Applied Biosystems). Real-time PCR conditions were 95˚C for 5 minutes, and 40 cycles of 95˚C for 15 seconds and 60˚C for 15 seconds, and 72˚C for 30 seconds.

### Confocal microscopic inspection.

Tumor-infiltrating CD8^+^ T cells were isolated from freshly digested tumor tissue using Mouse CD8a Positive Selection Kit (STEMCELL) and were seeded onto the cell slides, fixed with 4% paraformaldehyde (Sigma-Aldrich), and then permeabilized with 0.2% triton (Sigma-Aldrich) in 5% normal donkey serum (Jackson Immunoresearch). Cells were incubated with rabbit anti-HSP90 (CST, 4877, 1:50) and mouse anti-STAT5 (proteintech, 66459-1-Ig, 1:500) antibodies followed by incubation with FITC–conjugated anti-rabbit (1:200) and Texas red-conjugated anti-mouse (1:200) antibodies in 5% normal donkey serum in PBS. Slides were mounted with DAPI (Vector Laboratories Inc.). Images were then analyzed by Leica confocal microscopy.

### Coimmunoprecipitation and Western blot.

Cell extracts were prepared with lysis buffer (50 mM pH 7.5 Tris [Sangong Biotech], 150 mM NaCl [Sangong Biotech], 0.5% Triton X-100 [Sigma-Aldrich], 10% glycerol [Sangong Biotech], 2 mM EDTA [Invitrogen], 1 mM PMSF [Sigma-Aldrich], 20 mM, Protease Inhibitor Cocktail [Bimake], Phosphatase Inhibitor Cocktail [Bimake], and 2 mM DTT [BBI Solutions]). Dynabeads M-280 Sheep anti-Mouse IgG (Thermo Fisher Scientific) were incubated with RIG-I mouse monoclonal IgG (Santa Cruz, sc-376845), anti-Flag mouse monoclonal antibody (CST, 8146), or control IgG (Santa Cruz, sc-2025). Supernatants were then incubated with the preconjugated beads at 4°C overnight. The beads were sequentially washed 5 times with Co-IP lysis buffer. The bound protein was eluted with 2% SDS lysis buffer and boiled at 100˚C for 15 minutes. The proteins were then analyzed by Western blotting. Samples were diluted with 5 × SDS-PAGE Sample Loading Buffer (Beyotime) and denatured by heating to 100°C for 10 minutes. Equal amounts of protein samples were separated by 10% SDS-PAGE gels and transferred into PVDF membranes (0.45 μm, Millipore). Membranes were blocked for 1 hour at room temperature with 5% nonfat dry milk in Tris-buffered saline with 0.1% Tween-20 and then incubated with primary antibodies overnight at 4°C. The primary antibodies are listed below: mouse RIG-I mAb (Santa Cruz, sc-376845), mouse β-actin mAb (CST, 3700), rabbit Bcl2 mAb (CST, 3498), rabbit Bax mAb (CST, 147967), rabbit Bcl-xL mAb (CST, 2764), rabbit GZMA pAb (ABclonal, A6231), rabbit FLAG mAb (CST, 14793), rabbit HA mAb (CST, 3724), rabbit phospho-Stat5 (Tyr694) mAb (CST, 4322), rabbit Stat5 mAb (ABclonal, A5029), rabbit phospho-Stat3 (Tyr705) mAb (CST, 9145), rabbit Stat3 mAb (ABclonal, A1192), rabbit phospho-Stat1 mAb (CST, 9167), rabbit Stat1 mAb (CST, 14994), rabbit phospho-Irf3 mAb (CST, 29047), rabbit Irf3 mAb (CST, 4302), and rabbit GAPDH mAb (CST, 5174). Membranes were washed in TBS-T 3 times and incubated with horseradish peroxidase–conjugated secondary antibodies. Signals were detected with an Immobilon Western HRP substrate (Millipore) and visualized using Amersham Imager 600 System (GE Healthcare Bio-Sciences).

### T cell killing assay.

*Rig-I^+/+^* or *Rig-I^–/–^* naive CD8^+^ T cells were transfected with negative control, *Rig-I*-GFP, or *Stat5a*-shRNA-GFP and cultured overnight after 1-day of stimulation with precoated CD3/CD28 antibody. 5 × 10^4^ tumor cells were cultured in a 96-well plate and 1 day later, the indicated number of CD8^+^ T cells (listed in each figure) were reseeded and cocultured with tumor cells at 37°C under 5% CO_2_ in RPMI-1640 complete medium overnight. Cells were stained with PE conjugated CD8 antibody for 20 minutes to distinguish tumor and T cells and then APC conjugated Annexin V for 10 minutes according to the manufacturer’s instructions. The cells were examined by flow cytometry.

### RNA-Seq.

Fresh splenic naive CD8^+^ T cells were isolated from 6-week-old WT or KO mice and stimulated with plate-bounded anti-CD3 and anti-CD28 for 48 hours. Activated CD8^+^ T cells were used for total RNA extraction using Trizol (Life Technologies Corp.) and subjected to Illumina sequencing with paired-end 2 × 150 as the sequencing mode. The clean reads were mapped to the mouse genome (assembly GRCm38) using the HISAT2 software. Gene expression levels were estimated using FPKM, or fragments per kilobase of exon per million fragments mapped, by StringTie. Ballgown, a R package, was used to measure differential gene expression. The FDR control method was used to calculate the adjusted *P* values in multiple testing in order to evaluate the significance of the differences. Here, only genes with a *P*_adj_ <0.05 were used for subsequent analysis.

### Single-cell RNA-Seq data analysis.

Single-cell RNA-Seq counts were obtained from the Gene Expression Omnibus database with the accession number GSE108989 (41). Cells were divided into two high and low groups based on the median expression level of *RIG-I*. Genes with expression of greater than 0.1 in at least 2 samples in each group were selected for GSEA analysis. Gene sets were obtained from MSigDB and the Reactome database. GSEA (4.1.0) was used to determine whether the related gene sets were statistically enriched in groups with high or low *RIG-I* expression.

### Statistics.

The results are expressed as mean ± SEM. Statistics were performed using GraphPad Prism 9.0 or SPSS Statistics software. Unpaired or paired 2-tailed Student’s *t* tests, 1-Way ANOVA, and Turkey method for multiple comparisons, or 2-Way ANOVA was used to calculate *P* values where appropriate. Baseline characteristics of enrolled patients were simply described by frequency and percentage. Log-rank and multivariate Cox regression analysis were performed to investigate predictors of OS. OS was evaluated based on the expression of RIG-I by Kaplan-Meier analysis. *P* < 0.05 was considered to be statistically significant.

### Data and code availability.

The RNA-Seq data reported in this paper are available under accession number PRJNA 913263 (NCBI Trace and Short-Read Archive).

### Study approval.

Informed consent was obtained prior to participation from each healthy volunteer and patient. The study protocol was approved by the Clinical Research Ethics Committee of Ruijin Hospital (reference: 2017/61). Collection of human samples was approved by the Human Ethics Committee of Ruijin Hospital and was performed according to the principles of the Declaration of Helsinki. All animal procedures were approved by the IACUC of the Shanghai Jiao Tong University School of Medicine.

## Author contributions

XJ, JL, JZ, and HY conceived and designed the study. XJ, JL, and XW conducted experiments, handled data acquisition and interpretation, and wrote the first manuscript draft. CS, JZ, YS, and HY contributed to patient recruitment and the collection of biomaterials and clinical data. BX contributed to data analysis. JC, HG, and WZ provided the materials used in experiments. JZ and HY supervised study. All authors reviewed, edited, and approved the final version of the manuscript.

## Supplementary Material

Supplemental data

Supplemental data set 1

Supplemental data set 2

## Figures and Tables

**Table 1:**
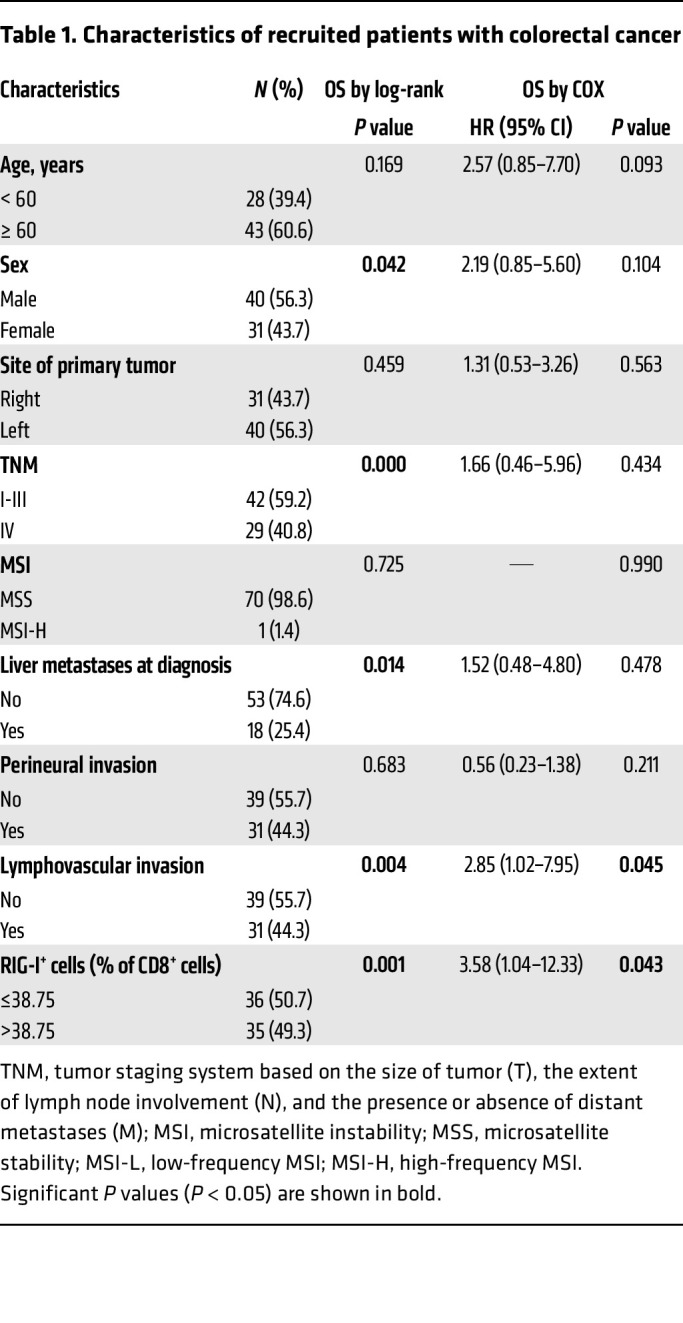
Characteristics of recruited patients with colorectal cancer

## Version 1 03/16/2023

In-Press Preview

## Version 2 05/01/2023

Electronic publication
