# Supplementary material for: Intrinsic RIG-I restrains STAT5 activation to modulate antitumor activity of CD8+ T cells
Source: J Clin Invest. 2023 May 1;133(9):e160790. doi: 10.1172/JCI160790 (PMC10145944; doi:10.1172/JCI160790)
Supplement: Supplemental data [file jci-133-160790-s188.pdf]

Figure S1

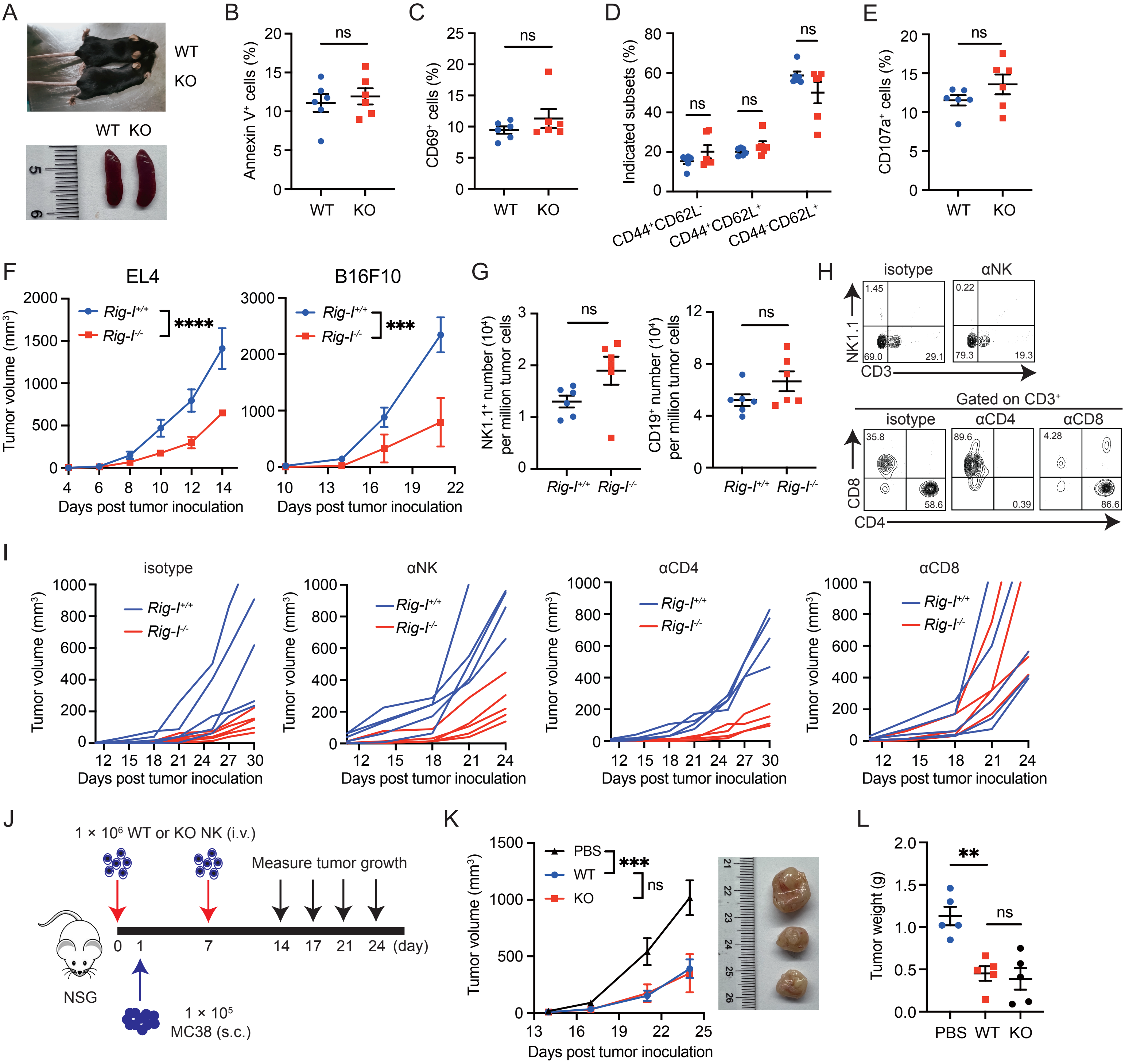
A
WT
KO
WT KO
B
Annexin V+ cells (%)
ns
WT KO
C
CD69+ cells (%)
ns
WT KO
D
Indicated subsets (%)
ns
ns
ns
CD44+CD62L-
CD44+CD62L+
CD44-CD62L+
E
CD107a+ cells (%)
ns
WT KO
F
EL4
B16F10
Tumor volume (mm3)
Rig-I+/+
Rig-I-/-
****
***
Days post tumor inoculation
G
NK1.1+ number (104) per million tumor cells
CD19+ number (104) per million tumor cells
ns
ns
Rig-I+/+ Rig-I-/-
H
isotype
αNK
NK1.1
CD3
1.45
69.0
29.1
0.22
79.3
19.3
Gated on CD3+
isotype
αCD4
αCD8
CD8
CD4
35.8
58.6
89.6
0.39
4.28
86.6
I
isotype
αNK
αCD4
αCD8
Tumor volume (mm3)
Rig-I+/+
Rig-I-/-
Days post tumor inoculation
J
1 × 106 WT or KO NK (i.v.)
Measure tumor growth
0 1 7 14 17 21 24 (day)
NSG
1 × 105 MC38 (s.c.)
K
Tumor volume (mm3)
PBS
WT
KO
***
ns
Days post tumor inoculation
L
Tumor weight (g)
**
ns
PBS WT KO

Figure S1. Tumor inhibitory effect of *Rig-I* knockout is attributed to CD8$^+$ T cells.

(A) Representative picture of 6-week-old *Rig-I*$^{+/+}$ (WT) or *Rig-I*$^{-/-}$ (KO) mice (upper panel) or spleens of 6-week-old WT or KO mice (bottom panel).

(B-E) The combined plots of flow cytometric analyses of Annexin V (B), CD69 (C), CD44 and CD62L (D) or CD107a (E) levels on splenic CD8$^+$ cells from WT or KO mice at 6 weeks old.

(F) 2 × 10^5 EL4 (left panel) or B16F10 cells (right panel) were subcutaneously inoculated into 6-week-old *Rig-I*$^{+/+}$ or *Rig-I*$^{-/-}$ mice and tumor volume was monitored for 2-3 weeks (n = 6 per group).

(G) Quantification of indicated subsets of dissociated MC38 tumor in *Rig-I*$^{+/+}$ or *Rig-I*$^{-/-}$ mice.

(H) Representative flow cytometric analyses of indicated proteins in peripheral blood from *Rig-I*$^{+/+}$ or *Rig-I*$^{-/-}$ mice on day 10.

(I) Tumor growth curve of individual *Rig-I*$^{+/+}$ or *Rig-I*$^{-/-}$ mouse inoculated with MC38 cells and treated with indicated antibody.

(J) 1 × 10^6 NK cells from spleens of *Rig-I*$^{+/+}$ or *Rig-I*$^{-/-}$ mice were intravenously transfused into NSG mice. Saline was used as a vehicle control. 1 × 10^5 MC38 cells were subcutaneously inoculated 1 day post NK cell injection and tumor volume was measured on day 14, 17, 21 and 24 (n = 5 per group).

(K) Tumor growth curve (left panel) and representative picture of tumor from each group on day 24 (right panel) are shown

(L) Combined plot of tumor weight on day 24.

Data are representative of two independent experiments and expressed as mean ± SEM. * $P < 0.05$ and ** $P < 0.01$ by unpaired Student's *t* test (B-E, L) or 2-way ANOVA (F, K).

Figure S2

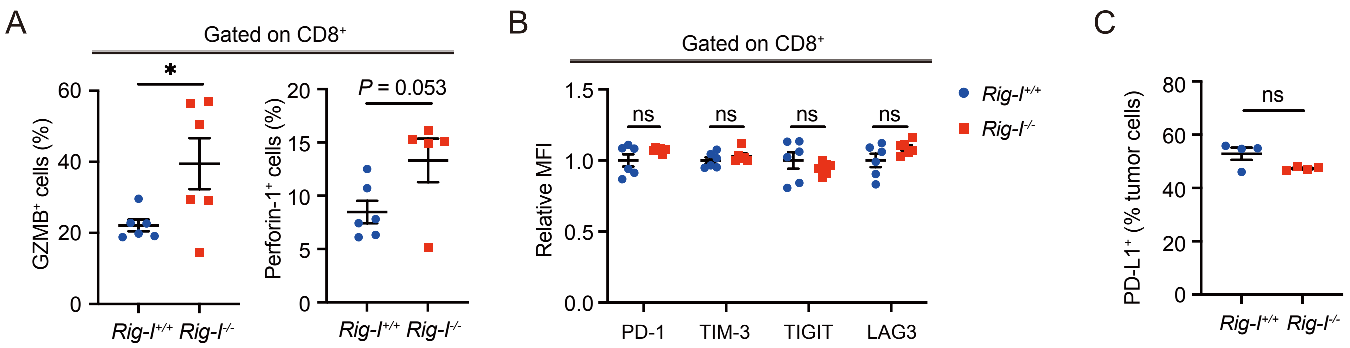

A
Gated on CD8+
*
P = 0.053
GZMB+ cells (%)
Perforin-1+ cells (%)
Rig-I+/+ Rig-I-/-
B
Gated on CD8+
Relative MFI
ns
PD-1
TIM-3
TIGIT
LAG3
Rig-I+/+
Rig-I-/-
C
PD-L1+ (% tumor cells)
ns
Rig-I+/+ Rig-I-/-

Figure S2. Evaluation of immunophenotype of *Rig-I* ablated $CD8^+$ T cells.

(A) The combined plots of flow cytometric analyses of granzyme B (GZMB) and perforin-1 are shown.

(B) The expression of inhibitory proteins in tumor-infiltrating $CD45^+CD8^+$ T cell of *Rig-I*$^{+/+}$ or *Rig-I*$^{-/-}$ mice were detected by flow cytometry and the relative mean fluorescence intensity was calculated.

(C) The proportion of PD-L1$^+$ cells in MC38 tumor cells of *Rig-I*$^{+/+}$ or *Rig-I*$^{-/-}$ mice subcutaneously inoculated tumor tissue was detected by flow cytometry.

Data are representative of two independent experiments and expressed as mean ± SEM. * $P < 0.05$ by unpaired Student's *t* test.

Figure S3

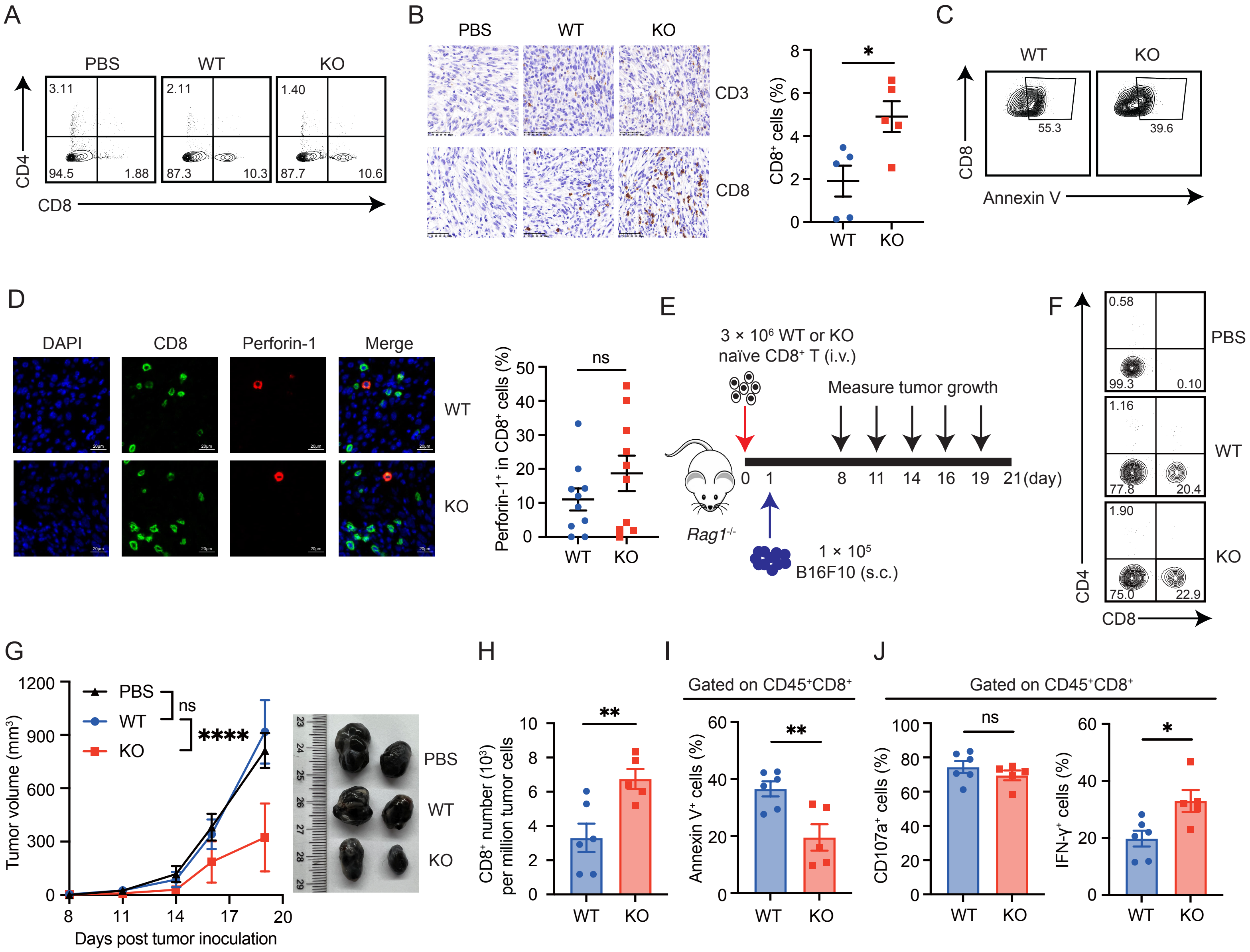

A
PBS
WT
KO
3.11
94.5
1.88
2.11
87.3
10.3
1.40
87.7
10.6
CD4
CD8
B
PBS
WT
KO
CD3
CD8
CD8+ cells (%)
WT
KO
C
WT
KO
55.3
39.6
CD8
Annexin V
D
DAPI
CD8
Perforin-1
Merge
WT
KO
Perforin-1+ in CD8+ cells (%)
ns
E
3 × 10^6 WT or KO
naïve CD8+ T (i.v.)
Measure tumor growth
0 1 8 11 14 16 19 21(day)
Rag1-/-
1 × 10^5
B16F10 (s.c.)
F
0.58
99.3
0.10
PBS
1.16
77.8
20.4
WT
1.90
75.0
22.9
KO
CD4
CD8
G
PBS
WT
KO
ns
****
Tumor volume (mm3)
Days post tumor inoculation
H
CD8+ number (10^3) per million tumor cells
I
Gated on CD45+CD8+
Annexin V+ cells (%)
J
Gated on CD45+CD8+
CD107a+ cells (%)
IFN-γ+ cells (%)

Figure S3. Engraftment, survival and effector function of adoptively transferred CD8$^+$ T cells.

(A) Representative flow cytometric analyses of CD4$^+$ and CD8$^+$ cells in spleens of *Rag1*$^{-/-}$ mice injected with PBS, *Rig-I*$^{+/+}$ or *Rig-I*$^{-/-}$ naïve CD8$^+$ T cells.

(B) Representative IHC staining images of indicated markers in tumor tissue (left panel) and the combined plot illustrating percentages of CD8$^+$ T cells of nucleated cells in one slide are shown (right panel, n = 5 per group).

(C) Representative flow cytometric analyses of Annexin V$^+$ cells in tumor-infiltrating CD8$^+$ T cells are shown.

(D) Immunofluorescence staining of tumor tissue was performed to detect CD8 and perforin-1. 10 random fields for each group were analyzed. Representative images (left panel) and combined plot (right panel) of the percentages of perforin-1$^+$ CD8$^+$ T cells are shown.

(E-F) 3 × 10^6 naïve CD8$^+$ T cells from spleens of *Rig-I*$^{+/+}$ or *Rig-I*$^{-/-}$ mice were intravenously transfused into *Rag1*$^{-/-}$ mice. Saline was used as a vehicle control. 1 × 10^5 B16F10 cells were subcutaneously inoculated 1 day post T cell injection and tumor volume was measured on day 8, 11, 14, 16 and 19 (n = 6 per group) (E). Representative flow cytometric analyses of CD4$^+$ and CD8$^+$ cells in peripheral blood of *Rag1*$^{-/-}$ mice on day 17 are shown (F).

(G) Tumor growth curve of each group (left panel) and representative picture of tumor on day 21 (right panel) are shown.

(H) $CD45^+CD8^+$ cell number was counted using flow cytometry.

(I-J) Annexin V, IFN-γ and CD107a were detected by flow cytometry. The percentage of Annexin V^+ cells (I) and IFN-γ^+ or $CD107a^+$ cells (J) in $CD45^+CD8^+$ cells are shown.

Data are representative of at least two independent experiments and expressed as mean ± SEM. * $P < 0.05$ and ** $P < 0.01$, by unpaired Student's *t* test (B, D, H-J) or 2-way ANOVA (G).

Figure S4

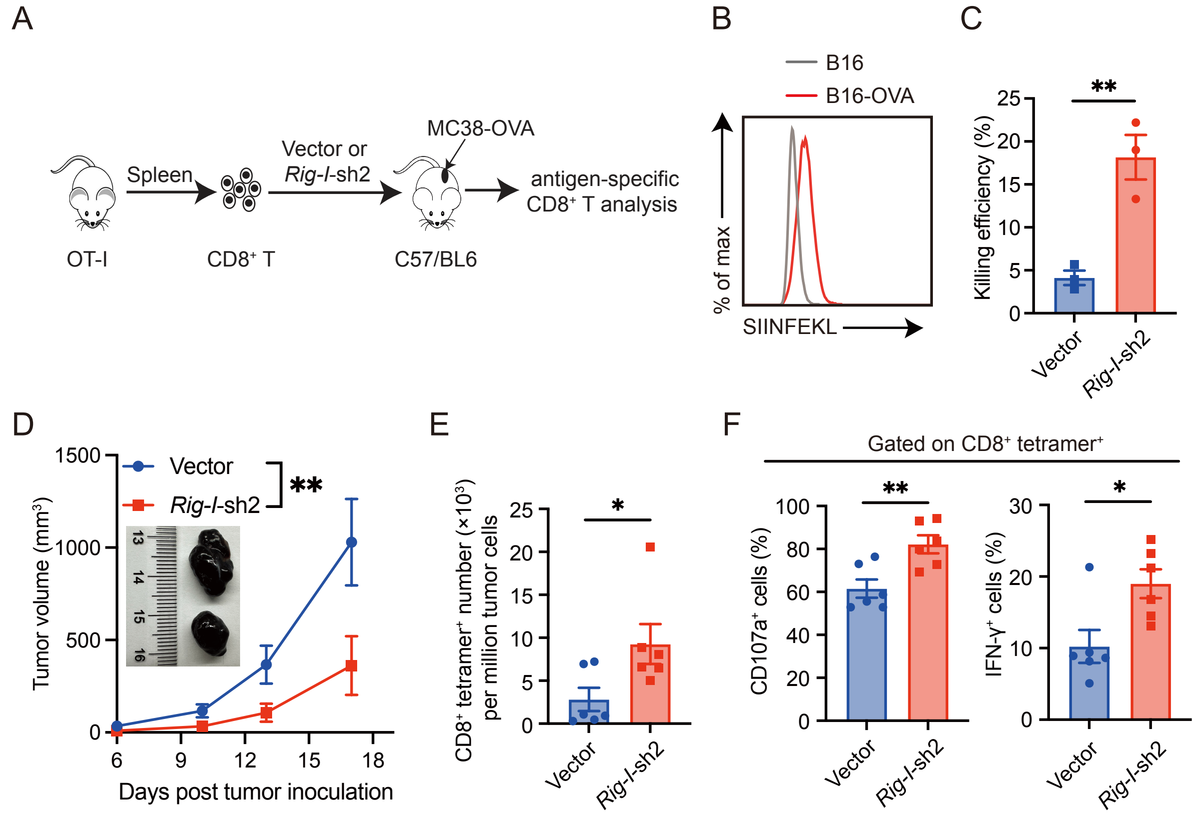
A
Spleen
Vector or
Rig-I-sh2
MC38-OVA
antigen-specific
CD8+ T analysis
OT-I
CD8+ T
C57/BL6
B
B16
B16-OVA
% of max
SIINFEKL
C
Killing efficiency (%)
**
Vector
Rig-I-sh2
D
Vector
Rig-I-sh2
**
Tumor volume (mm3)
Days post tumor inoculation
E
CD8+ tetramer+ number (×103)
per million tumor cells
*
Vector
Rig-I-sh2
F
Gated on CD8+ tetramer+
CD107a+ cells (%)
**
IFN-γ+ cells (%)
*
Vector
Rig-I-sh2

Figure S4. RIG-I inhibits survival and effector function of transferred antigen-specific CD8$^+$ T cells.

(A) 2 × 10^5 MC38-OVA tumor cells were subcutaneously inoculated in WT C57 mice. CD8$^+$ T cells isolated from the spleens of OT-I mice were retrovirally transfected with vector or *Rig-I*-shRNA-2, and a total of 2 × 10^6 infected OT-I cells were transferred into mice bearing MC38-OVA tumor (n = 5 per group) when the tumor was visible.

(B) The presentation of OVA-derived SIINFEKL peptide by H-2Kb in B16F10 or B16-OVA cells were analyzed by flow cytometry.

(C) Vector or *Rig-I*-shRNA-2 transfected CD8$^+$ T cells from spleens of OT-I mice were co-cultured with B16-OVA cells at 1:1 ratio overnight and killing efficiency were analyzed by flow cytometry.

(D) 3 × 10^5 B16-OVA tumor cells were subcutaneously inoculated in WT C57 mice. CD8$^+$ T cells isolated from the spleens of OT-I mice were retrovirally transfected with vector or *Rig-I*-shRNA-2, and a total of 3 × 10^6 infected OT-I cells were transferred into mice bearing B16-OVA tumor (n = 6 per group) when the tumor was visible. Tumor growth curve and representative picture of tumors retrieved from mice on day 18 are shown.

(E-F) Tumors were extracted 18 days later after tumor inoculation for tumor-infiltrating CD8$^+$ tetramer$^+$ T cell analysis. CD8$^+$ tetramer$^+$ T cell number was

counted (E) and the percentages of IFN-γ^+ or CD107a$^+$ cells (F) of antigen-specific CD8$^+$ T cells were analyzed by flow cytometry.

Data are representative of at least two independent experiments and expressed as mean ± SEM. * $P < 0.05$ and ** $P < 0.01$, by unpaired Student's *t* test (C, E, F) or 2-way ANOVA (D).

Figure S5

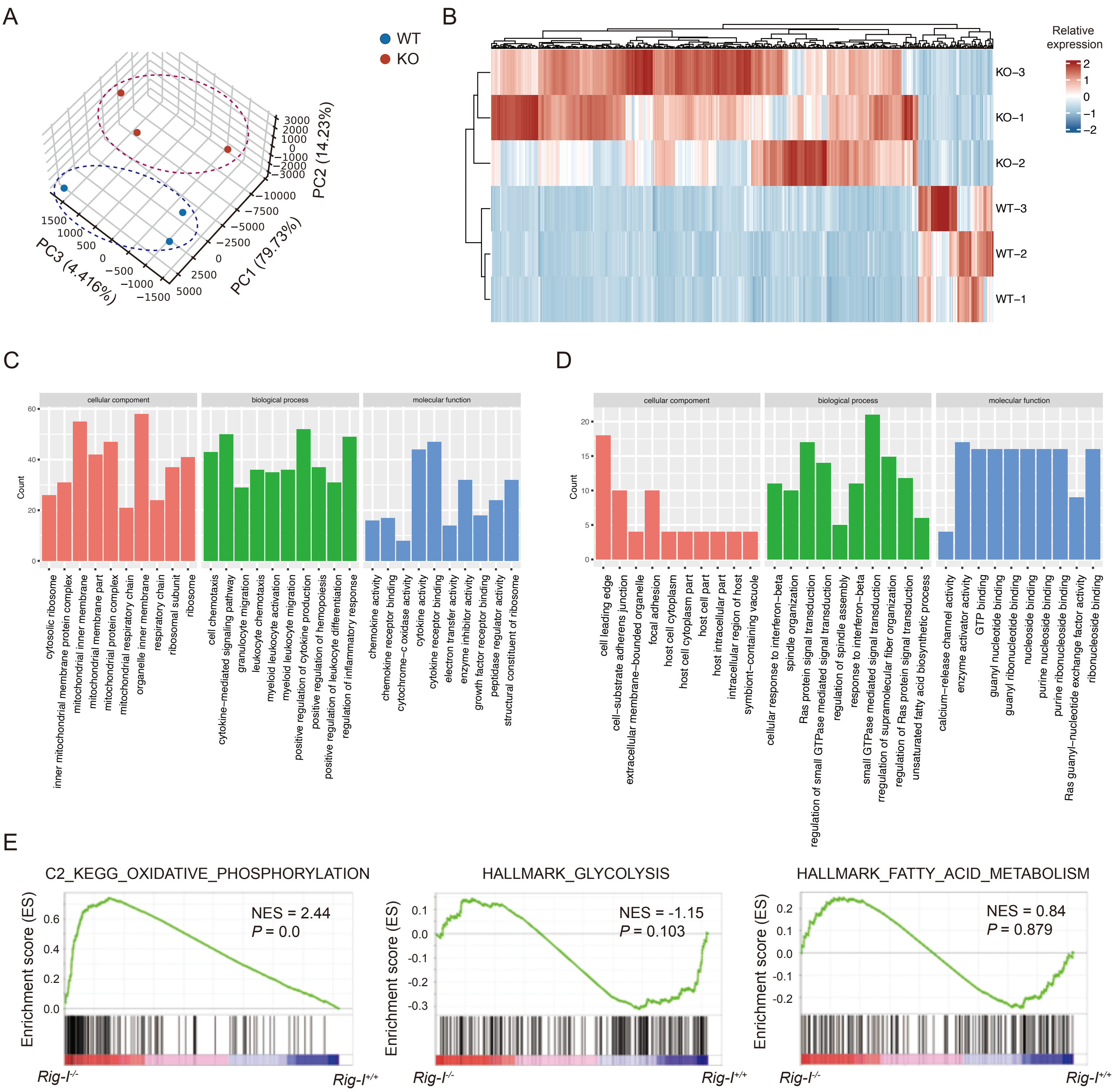
A
WT
KO
PC1 (79.73%)
PC2 (14.23%)
PC3 (4.416%)
B
Relative expression
KO−3
KO−1
KO−2
WT−3
WT−2
WT−1
C
cellular compoment
biological process
molecular function
Count
cytosolic ribosome
inner mitochondrial membrane protein complex
mitochondrial inner membrane
mitochondrial membrane part
mitochondrial protein complex
mitochondrial respiratory chain
organelle inner membrane
respiratory chain
ribosomal subunit
ribosome
cell chemotaxis
cytokine−mediated signaling pathway
granulocyte migration
leukocyte chemotaxis
myeloid leukocyte activation
myeloid leukocyte migration
positive regulation of cytokine production
positive regulation of hemopoiesis
positive regulation of leukocyte differentiation
regulation of inflammatory response
chemokine activity
chemokine receptor binding
cytochrome−c oxidase activity
cytokine activity
cytokine receptor binding
electron transfer activity
enzyme inhibitor activity
growth factor receptor binding
peptidase regulator activity
structural constituent of ribosome
D
cellular compoment
biological process
molecular function
Count
cell leading edge
cell−substrate adherens junction
extracellular membrane−bounded organelle
focal adhesion
host cell cytoplasm
host cell cytoplasm part
host cell part
host intracellular part
intracellular region of host
symbiont−containing vacuole
cellular response to interferon−beta
spindle organization
Ras protein signal transduction
regulation of small GTPase mediated signal transduction
regulation of spindle assembly
response to interferon−beta
small GTPase mediated signal transduction
rregulation of supramolecular fiber organization
regulation of Ras protein signal transduction
unsaturated fatty acid biosynthetic process
calcium−release channel activity
enzyme activator activity
GTP binding
guanyl nucleotide binding
guanyl ribonucleotide binding
nucleoside binding
purine nucleoside binding
purine ribonucleoside binding
Ras guanyl−nucleotide exchange factor activity
ribonucleoside binding
E
C2_KEGG_OXIDATIVE_PHOSPHORYLATION
NES = 2.44
P = 0.0
HALLMARK_GLYCOLYSIS
NES = -1.15
P = 0.103
HALLMARK_FATTY_ACID_METABOLISM
NES = 0.84
P = 0.879
Enrichment score (ES)
Rig-I-/-
Rig-I+/+

Figure S5. Rig-I ablation broadly reprograms the expressional profiles of activated CD8+ T cells.

(A-E) Naïve *Rig-I*$^{+/+}$ or *Rig-I*$^{-/-}$ CD8^{+} T cells were cultured under the stimulation of anti-CD3/CD28 for 48 hours and were submitted for RNA sequencing. Principal component (PC) analysis of genes detected in 3 replicates of *Rig-I*$^{+/+}$ or *Rig-I*$^{-/-}$ *in vitro* activated CD8^{+} T cells (A). Heatmap showing the top 500 differentially expressed genes from bulk RNA sequencing of *in vitro* activated *Rig-I*$^{+/+}$ and *Rig-I*$^{-/-}$ CD8^{+} T cells (B). GO pathways significantly up-regulated (C) or down-regulated (D) in *in vitro* activated *Rig-I*$^{-/-}$ CD8^{+} T cells compared to *Rig-I*$^{+/+}$ counterpart. GSEA analysis of indicated pathways is showed (E).

Figure S6

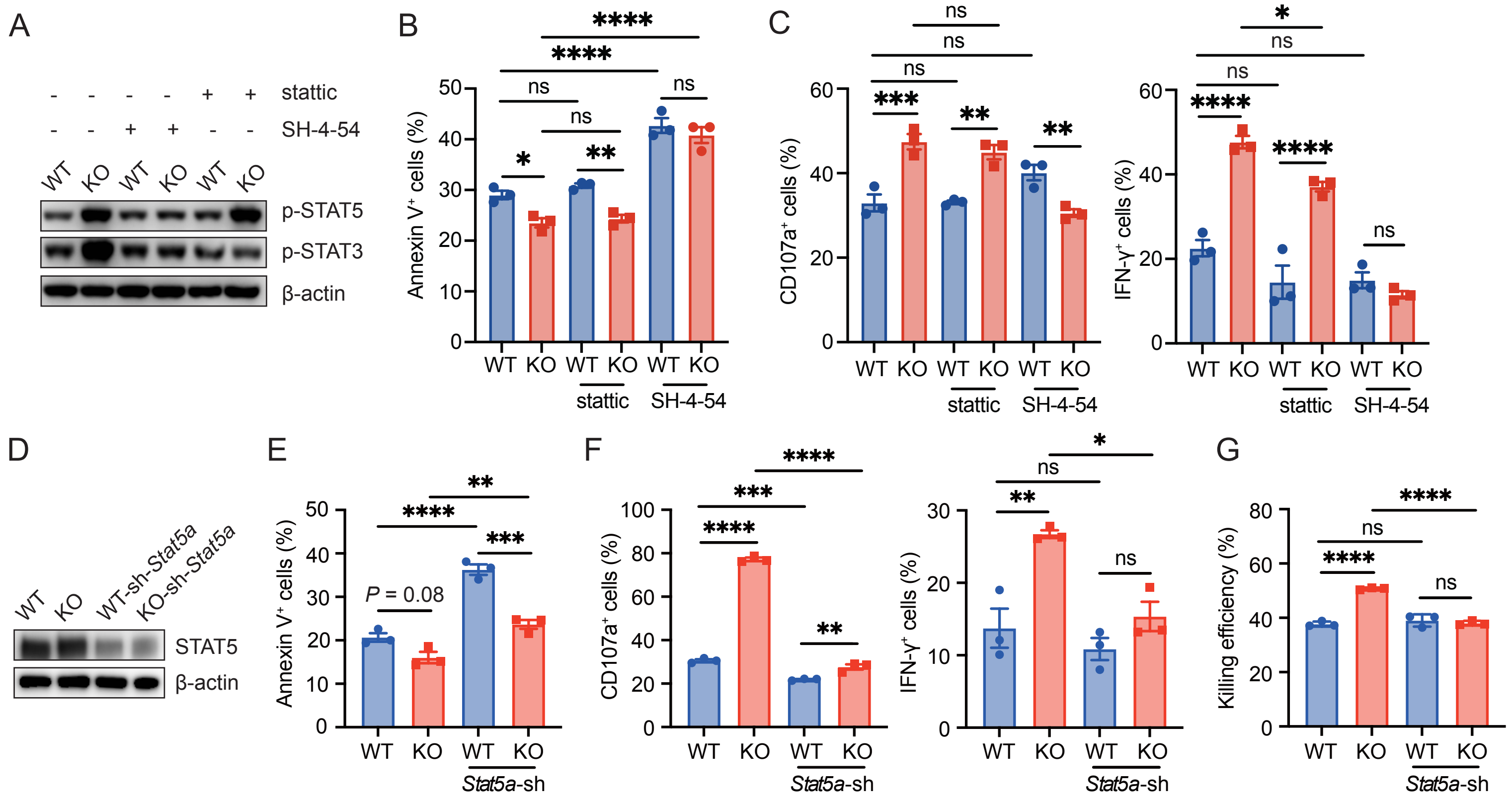
A
B
C
D
E
F
G
stattic
SH-4-54
WT KO WT KO WT KO
p-STAT5
p-STAT3
β-actin
Annexin V⁺ cells (%)
CD107a⁺ cells (%)
IFN-γ⁺ cells (%)
ns
*
**
****
***
WT KO WT KO WT KO
stattic
SH-4-54
WT
KO
WT-sh-Stat5a
KO-sh-Stat5a
STAT5
β-actin
P = 0.08
Stat5a-sh
Killing efficiency (%)

Figure S6. RIG-I restrains CD8$^+$ T cell survival and cytotoxicity by blocking STAT5 activation.

(A) Western blotting analyses of p-STAT5 (Tyr694) or p-STAT3 (Tyr705) to determine the efficiency of stattic and SH-4-54.

(B-C) Naïve CD8$^+$ T cells from spleens of *Rig-I*$^{+/+}$ or *Rig-I*$^{-/-}$ mice were stimulated by anti-CD3/CD28 for 1 day prior to the treatment of stattic (10 nM) or SH-4-54 (25 nM) for another 2 days. Annexin V, CD107a and IFN-γ levels were analyzed by flow cytometry. The percentages of Annexin V$^+$ cells (B) and CD107a$^+$ or IFN-γ$^+$ cells (C) of CD8$^+$ T cells are shown.

(D) Western blotting analysis of STAT5 protein level to determine the efficiency of *Stat5a*-shRNA.

(E-F) Naïve *Rig-I*$^{+/+}$ or *Rig-I*$^{-/-}$ CD8$^+$ T cells stimulated by anti-CD3/CD28 for 1 day before retrovirally transfected with control vector or *Stat5a*-shRNA and cultured for another 2 days. Annexin V, CD107a and IFN-γ were analyzed by flow cytometry. The percentage of Annexin V$^+$ cells (E) and CD107a$^+$ or IFN-γ$^+$ (F) are shown.

(G) Activated *Rig-I*$^{+/+}$ or *Rig-I*$^{-/-}$ CD8$^+$ T cells or CD8$^+$ T cells transfected with *Stat5a*-shRNA were co-cultured with MC38 cells at 10:1 ratio for 1 day *in vitro*. Killing efficiency was analyzed by flow cytometry.

Data are representative of three independent experiments and expressed as mean ± SEM. * $P < 0.05$, ** $P < 0.01$, *** $P < 0.001$, **** $P < 0.0001$, by 1-way ANOVA.

Figure S7

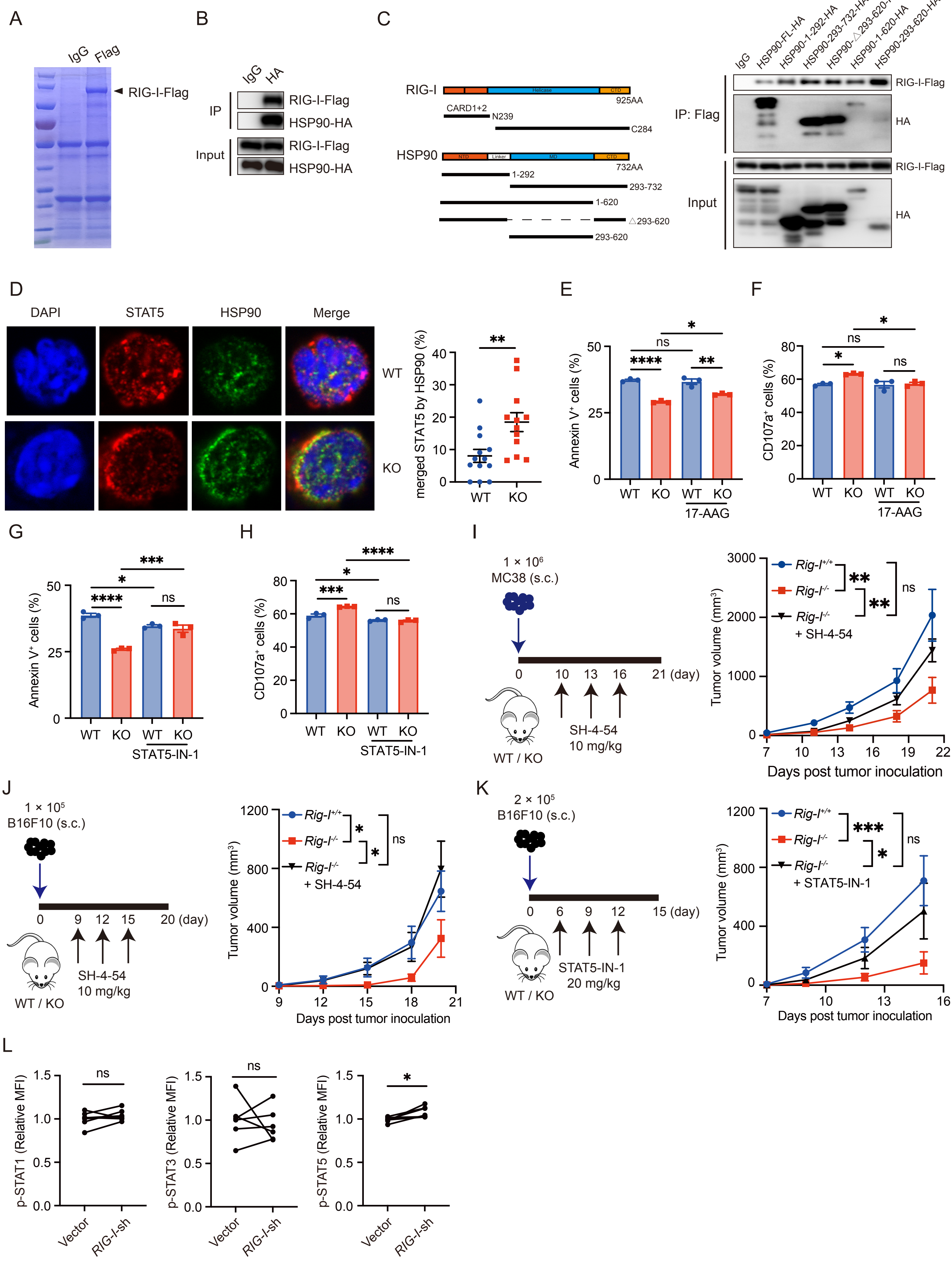
A
IgG
Flag
RIG-I-Flag
B
IgG
HA
IP
Input
RIG-I-Flag
HSP90-HA
RIG-I-Flag
HSP90-HA
C
RIG-I
925AA
CARD1+2
N239
C284
HSP90
732AA
1-292
293-732
1-620
△293-620
293-620
IgG
HSP90-FL-HA
HSP90-1-292-HA
HSP90-293-732-HA
HSP90-△293-620-HA
HSP90-1-620-HA
HSP90-293-620-HA
IP: Flag
Input
RIG-I-Flag
HA
D
DAPI
STAT5
HSP90
Merge
WT
KO
merged STAT5 by HSP90 (%)
E
Annexin V+ cells (%)
17-AAG
F
CD107a+ cells (%)
G
STAT5-IN-1
H
I
1 × 10^6
MC38 (s.c.)
SH-4-54
10 mg/kg
WT / KO
21 (day)
Rig-I+/+
Rig-I-/-
Rig-I-/- + SH-4-54
Tumor volume (mm3)
Days post tumor inoculation
J
1 × 10^5
B16F10 (s.c.)
20 (day)
K
2 × 10^5
B16F10 (s.c.)
STAT5-IN-1
20 mg/kg
15 (day)
Rig-I-/- + STAT5-IN-1
L
p-STAT1 (Relative MFI)
p-STAT3 (Relative MFI)
p-STAT5 (Relative MFI)
Vector
RIG-I-sh
ns
*

Figure S7. RIG-I deficiency inhibits tumor growth by dampening HSP90-mediated protective effect on STAT5.

(A) Lysates from 293T cells transfected with Flag-tagged RIG-I plasmid were subjected to immunoprecipitation with IgG or Flag antibody. Coomassie blue staining of immunoprecipitated protein is shown.

(B) Immunoblot analysis of RIG-I and HSP90 interactions in 293T cells transfected with RIG-I-Flag and HSP90-HA plasmid.

(C) Schematic of critical domains of full-length RIG-I or HSP90. The constructs of truncated mutants of RIG-I or HSP90 are shown underneath (left panel). Lysates from 293T cells co-transfected with plasmids encoding Flag-tagged RIG-I and the indicated HA-HSP90 mutants were subjected to immunoprecipitation with Flag antibody. IP lysates were then analyzed by western blotting with indicated antibody (right panel).

(D-H) *Rig-I*$^{+/+}$ or *Rig-I*$^{-/-}$ tumor-infiltrating CD8^{+} T cells were isolated from freshly digested MC38 tumor tissue. (D) Isolated cells were co-stained by HSP90 antibody (green) and STAT5 antibody (red). DAPI was used to visualize the nuclei (blue). 12-13 fields and total cells (about 200 cells for each group) were counted. Representative images (left panel) and the proportion of cells with HSP90 merged with STAT5 (right panel) are shown. (E-H) Isolated cells were

cultured with 17-AAG (10 nM) or STAT5-IN-1 (20 μM) for 4 hours and Annexin V^+ or $CD107a^+$ $CD8^+$ T cells were detected by flow cytometry.

(I) *Rig-I*$^{+/+}$ or *Rig-I*$^{-/-}$ mice were subcutaneously inoculated with 1 × 10^6 MC38 cells and were treated with SH-4-54 (10 mg/kg) on day 10, 13 and 16. Schematic diagram (left panel) and tumor growth curve (right panel) are shown.

(J-K) *Rig-I*$^{+/+}$ or *Rig-I*$^{-/-}$ mice were subcutaneously inoculated with 1 × 10^5 (J) or 2 × 10^5 (K) B16F10 cells and were treated with SH-4-54 (10mg/kg, J) or STAT5-IN-1 (20 mg/kg, K) every 3 days for 3 times when the tumor was visible (n = 5-6 per group). Schematic diagram (left panel) and tumor growth curve (right panel) are shown.

(L) $CD8^+$ T cells sorted from human PBMCs were stimulated by anti-CD3/CD28 1 day prior to retroviral transfection of NC-GFP (Vector) or *RIG-I*-shRNA-GFP (*RIG-I*-sh) and cultured for 2 more days. Mean fluorescence intensity of p-STAT1, p-STAT3 or p-STAT5 of GFP^+ cells were analyzed by flow cytometry.

Data are representative of at least two independent experiments and expressed as mean ± SEM. * $P < 0.05$, ** $P < 0.01$, *** $P < 0.001$, *** $P < 0.0001$, by unpaired Student's t test (D), 1-way ANOVA (E-H), 2-way ANOVA (I-K) or paired Student's t test (L).

Figure S8

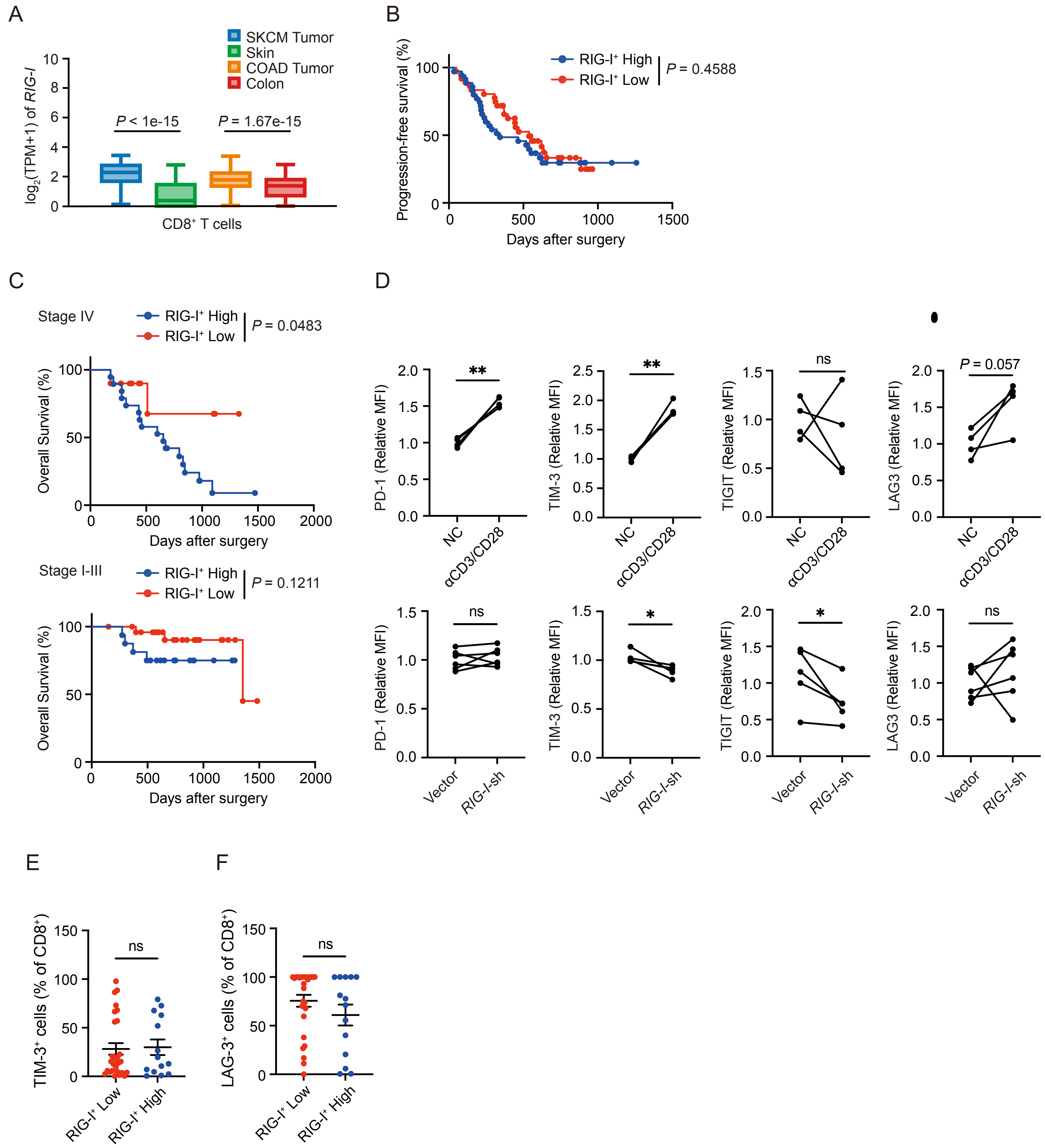
A
SKCM Tumor
Skin
COAD Tumor
Colon
log2(TPM+1) of RIG-I
P < 1e-15
P = 1.67e-15
CD8+ T cells
B
Progression-free survival (%)
RIG-I+ High
RIG-I+ Low
P = 0.4588
Days after surgery
C
Stage IV
RIG-I+ High
RIG-I+ Low
P = 0.0483
Overall Survival (%)
Days after surgery
Stage I-III
RIG-I+ High
RIG-I+ Low
P = 0.1211
Overall Survival (%)
Days after surgery
D
PD-1 (Relative MFI)
TIM-3 (Relative MFI)
TIGIT (Relative MFI)
LAG3 (Relative MFI)
**
**
ns
P = 0.057
NC
αCD3/CD28
ns
*
*
ns
Vector
RIG-I-sh
E
TIM-3+ cells (% of CD8+)
ns
RIG-I+ Low
RIG-I+ High
F
LAG-3+ cells (% of CD8+)
ns
RIG-I+ Low
RIG-I+ High

Figure S8. RIG-I level elevates in tumor-infiltrating CD8$^+$ T cells compared with CD8$^+$ T cells in normal tissues.

(A) *RIG-I* expression in CD8$^+$ T cells from indicated tissues. Data drawn from GEPIA2021 (http://gepia2021.cancer-pku.cn) using CIBERSORT deconvolution tool.

(B) Kaplan-Meier plots depicting progression-free survival of RIG-I$^+$ CD8$^+$ low group or RIG-I$^+$ CD8$^+$ high group are shown.

(C) Kaplan-Meier plots depicting overall survival from RIG-I$^+$ CD8$^+$ low group or RIG-I$^+$ CD8$^+$ high group of stage IV (upper panel) or stage I-III (bottom panel) are shown.

(D) CD8$^+$ T cells sorted from human PBMCs were stimulated by anti-CD3/CD28 for 2 days (upper panels). CD8$^+$ T cells sorted from human PBMCs were stimulated by anti-CD3/CD28 1 day prior to retroviral transfection of NC-GFP (Vector) or *RIG-I*-shRNA-GFP (*RIG-I*-sh) and cultured for 2 more days (bottom panels). Relative mean fluorescence intensity of inhibitory proteins (PD-1, TIM-3, TIGIT and LAG3) of GFP$^+$ cells were analyzed by flow cytometry.

(E-F) Slides of tumor tissue from colorectal carcinoma patients were co-stained by TIM-3 or LAG-3, RIG-I and CD8 antibody. DAPI was used to visualize the nuclei. The proportion of TIM-3$^+$ or LAG-3$^+$ cells of CD8$^+$ cells from RIG-I$^+$ CD8$^+$ low group or RIG-I$^+$ CD8$^+$ high group are shown.

Data are representative of two independent experiments (D). Data are expressed as mean ± SEM (E, F). * $P < 0.05$, ** $P < 0.01$, *** $P < 0.001$, *** $P < 0.0001$, by log-rank test (B, C), paired (D) or unpaired Student's *t* test (E, F).
